# Three-dimensional imaging of sulfur chemical states in polymers with micrometer thickness using sulfur *K*-edge ptychographic tomography

**DOI:** 10.1038/s41598-026-52630-4

**Published:** 2026-05-16

**Authors:** Yuhei Sasaki, Nozomu Ishiguro, Masaki Abe, Shuntaro Takazawa, Naru Okawa, Mihiro Ikenaga, Fusae Kaneko, Yukio Takahashi

**Affiliations:** 1 International Center for Synchrotron Radiation Innovation Smart (SRIS), Tohoku University, 468-1 Aramaki Aza-Aoba, Aoba-Ku, Sendai, 980-8572 Japan; 2 RIKEN SPring-8 Center, 1-1-1 Kouto, Sayo-Cho, Sayo-Gun, Hyogo, 679-5148 Japan; 3 Department of Metallurgy, Graduate School of Engineering, Tohoku University, 6-6-2 Aoba-Yama, Aoba-Ku, Sendai, 980-8579 Japan; 4 Institute of Multidisciplinary Research for Advanced Materials (IMRAM), Tohoku University, 2-1-1 Katahira, Aoba-Ku, Sendai, 980-8577 Japan; 5 Sumitomo Rubber Industries, Ltd., 2-1-1 Tsutsui, Chuo, Kobe, Hyogo 651-0071 Japan; 6 Institute for Materials Research, Tohoku University, 2-1-1 Katahira, Aoba-Ku, Sendai, 980-8577 Japan

**Keywords:** Chemistry, Materials science, Nanoscience and technology, Optics and photonics, Physics

## Abstract

Three-dimensional visualization of chemical bonding states in light-element materials remains challenging, particularly for micrometer-thick specimens, due to strong X-ray absorption and limited photon flux in the tender X-ray regime. Here three-dimensional chemical-state imaging of sulfur in sulfurized poly(n-butyl methacrylate) (SPBMA) samples up to ~ 3.3 µm thick is demonstrated using ptychographic X-ray computed tomography at the sulfur *K*-edge. A high-resolution ptychographic CT system at the NanoTerasu BL10U beamline enables quantitative mapping of electron density, sulfur concentration, and spectral metrics derived from multi-energy imaging, that serve as proxies for sulfur–sulfur and sulfur–carbon bonding states, with ~ 80 nm spatial resolution. Multi-energy volumetric analysis using four carefully chosen photon energies reveals pronounced spatial heterogeneity in sulfur bonding, distinguishing sulfur–sulfur and sulfur–carbon bonds that are inaccessible from spatially averaged spectra. This study establishes sulfur *K*-edge ptychographic tomography as a potential platform for three-dimensional chemical-state imaging of light-element materials, enabling new nanoscale investigations of functional polymers and energy-related materials.

**Supplementary Information:**

The online version contains supplementary material available at 10.1038/s41598-026-52630-4.

## Introduction

Three-dimensional imaging of chemical bonding states in light-element materials is crucial for understanding the structure–function relationships in functional polymers, energy-storage materials, and biological systems. However, achieving nanoscale three-dimensional chemical-state imaging in micrometer-thick samples remains challenging, particularly at the sulfur *K*-edge, due to strong X-ray absorption and limited photon flux in the tender X-ray regime (approximately 2000–5000 eV). Sulfur plays a key role in a wide range of materials, including energy-storage systems, biological tissues, and sulfur-crosslinked polymers^1–4^, where both the chemical states (e.g., S–S and S–C bonding) and their three-dimensional spatial distributions govern macroscopic properties. Despite this importance, existing techniques such as confocal Raman microscopy and electron microscopy^5,6^ are limited by insufficient penetration depth, restricted field of view, or lack of phase sensitivity, hindering quantitative three-dimensional analysis in micrometer-thick samples.

X-ray absorption spectroscopy (XAS) enables element-selective analysis of electronic structure and chemical states—including oxidation state, local coordination environment, and chemical bonding—and has been extended to spatially resolved imaging using scanning X-ray microscopy^7–9^. In these approaches, the spatial resolution is primarily determined by the focused beam size. Scanning X-ray fluorescence microscopy has been widely used for imaging sulfur species^10,11^; however, quantitative interpretation is often complicated by self-absorption and saturation effects, particularly in micrometer-thick or compositionally heterogeneous samples. These limitations become more severe at the sulfur *K*-edge due to strong absorption and limited photon flux. Moreover, these methods lack phase sensitivity to electron-density variations, which is critical for visualizing light-element structures. Differential phase contrast imaging using segmented detectors^12^ can also provide phase-contrast information in scanning X-ray microscopy; however, its spatial resolution is generally limited by the focused probe size. As a result, conventional scanning X-ray microscopy remains insufficient for comprehensive three-dimensional analysis of structure, composition, and chemical states in micrometer-thick materials composed of light elements, such as polymers.

To address the limitations of conventional scanning X-ray microscopy, X-ray ptychography has emerged as a robust coherent imaging method. In this approach, a sample undergoes scanning across overlapping positions of a coherent X-ray beam, and phase-retrieval algorithms are applied to far-field diffraction patterns to reconstruct both the sample image and the incident wavefront^13,14^. Unlike scanning X-ray microscopy, the spatial resolution in X-ray ptychography is determined by the maximum scattering angle of the diffraction patterns rather than by the probe size. X-ray ptychography yields quantitative phase images proportional to the projected electron density and absorption images that correspond to X-ray attenuation in the sample. Extension to ptychographic X-ray computed tomography (PXCT) enables three-dimensional imaging by capturing projection images while rotating the sample^15–17^. Additionally, multi-energy PXCT facilitates three-dimensional chemical-state analysis by leveraging resonant dispersion effects^18–20^. To date, PXCT has found successful applications in various systems such as catalysts, battery electrodes, and semiconductor materials^21–25^.

Despite the successful application of PXCT to a wide range of materials, X-ray ptychography near the sulfur *K*-edge remains challenging. The feasibility of sulfur *K*-edge ptychographic imaging was first demonstrated at the SPring-8 BL27SU beamline^26^, where sulfur-containing polymers were imaged with a spatial resolution of ~ 200 nm using zone-plate-based optics^27^. However, achieving high-resolution PXCT at the sulfur *K*-edge has been fundamentally limited by low photon flux in the tender X-ray energy range and detector performance. In particular, the dynamic range and detection efficiency of X-ray detectors become critical at these energies, where commonly used detectors often exhibit reduced sensitivity. To overcome these limitations, a high-resolution PXCT system combining total-reflection focusing mirrors with a fast, low-noise CITIUS detector was developed at the BL10U beamline of NanoTerasu^28–30^.

In this work, three-dimensional chemical-state imaging of sulfur in micrometer-thick polymers is demonstrated using PXCT at the sulfur *K*-edge. This approach enables quantitative reconstruction of composition-related metrics in three dimensions, including electron density, sulfur concentration, and spectral indicators that serve as proxies for sulfur-bonding states, with a spatial resolution of ~ 80 nm. Multi-energy PXCT allows volumetric separation of spatial variations in sulfur–sulfur and sulfur–carbon bonding configurations. Moreover, the spatial variations in composition-related metrics within the particles show patterns similar to projection-based results obtained by spectroscopic ptychography. These results establish sulfur *K*-edge ptychographic tomography as a robust approach for three-dimensional chemical-state imaging of light-element materials.

## Workflow of sulfur *K*-edge ptychographic X-ray computed tomography

**Fig. 1:**
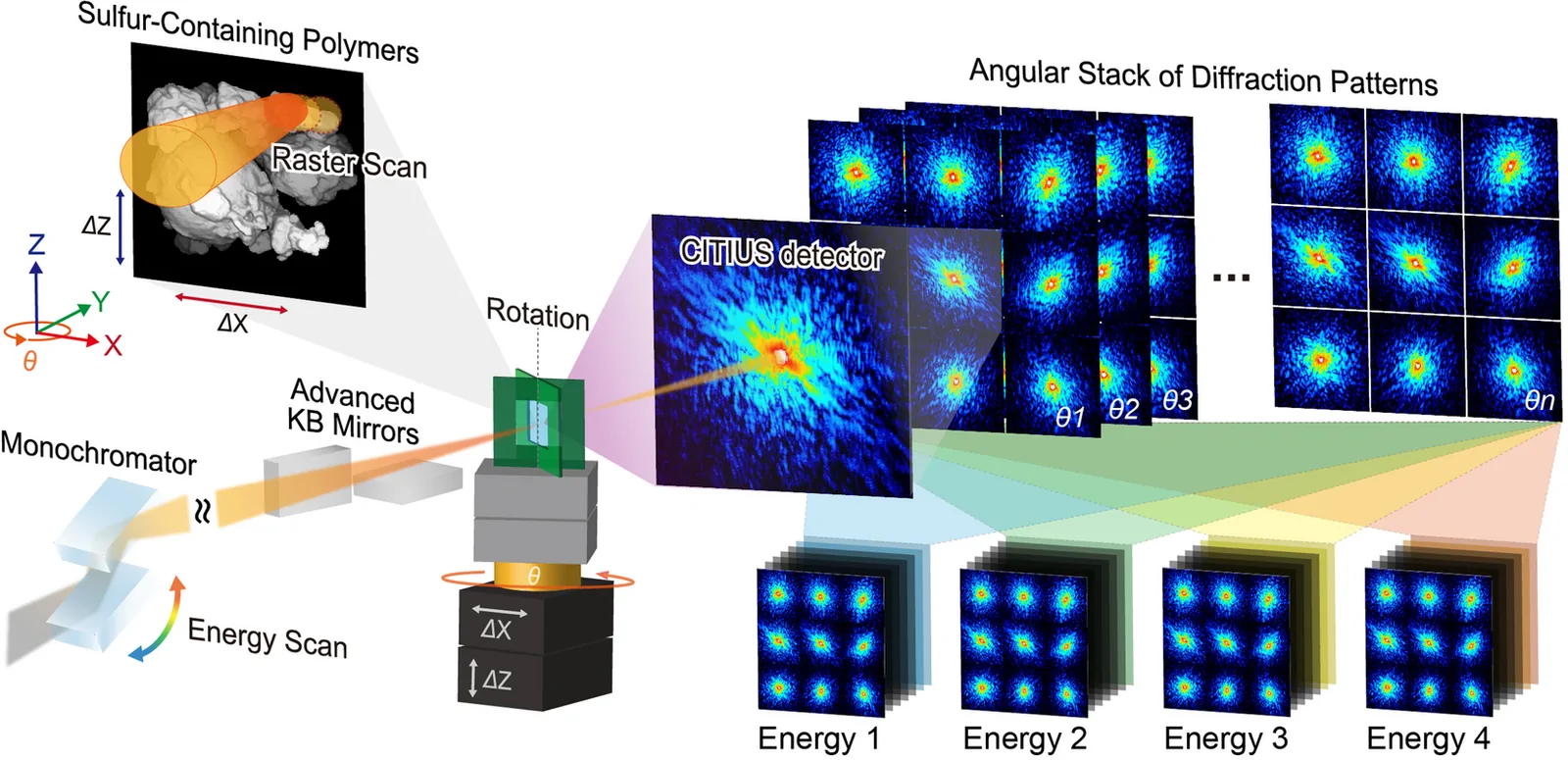
Schematic of ptychographic X-ray computed tomography measurements near the sulfur *K*-edge.

The sample used for sulfur *K*-edge PXCT was sulfurized poly(*n*-butyl methacrylate) (SPBMA), a sulfur–polymer composite similar to sulfurized polyacrylonitrile^31^. SPBMA is known to exhibit spatial heterogeneity in sulfur concentration and bonding configurations. It primarily consists of carbon, oxygen, and sulfur. Previous studies have suggested spatially heterogeneous distributions of sulfur concentration as well as sulfur–sulfur (S–S) and sulfur–carbon (S–C) bonding within individual particles^27^. To validate the characteristics of the prepared samples and ensure consistency with prior reports, the average composition and chemical states were examined using scanning electron microscopy with energy-dispersive X-ray spectroscopy (SEM–EDX) and XAS in the conversion electron yield (CEY) mode (Supplementary Notes 1 and 2). These measurements confirmed that the samples were comparable to those reported previously^27^. Synchrotron radiation experiments were conducted at the BL10U beamline of NanoTerasu using advanced Kirkpatrick–Baez (AKB) focusing optics^29,30^. The measurement system was slightly modified from the previously reported design (Methods and Supplementary Note 3). Figure 1 schematically illustrates the experimental setup. To enable ptychographic measurements in transmission geometry, the samples were supported on thin silicon nitride membranes. For each projection, diffraction patterns were acquired by raster scanning the sample with a step size of 500 nm over an array of 16 × 14 scan positions. The probe size was approximately 1.48 μm (H) × 1.28 μm (V). PXCT scans were performed at each energy while rotating the sample over an angular range of ± 84°. A total of 113 equally spaced projection angles were used. Measurements were conducted at four energies near the sulfur *K-*edge: 2500 eV (above the absorption edge), 2463 eV (below the absorption edge), 2470.7 eV (near the S–S bonding peak), and 2469.6 eV (near the S–C bonding peak). Phase-retrieval calculations were utilized to reconstruct the sample images from the diffraction patterns. SPMBA projection images were reconstructed using a refractive index-based ptychographic iterative engine with a two-mode mixed-state model and position correction^32–34^. Three-dimensional reconstruction with a voxel size of 25 nm was performed from angular stacks of the phase shift and absorption images using the simultaneous iterative reconstruction technique with a non-negativity constraint^35^.

## PXCT of sulfur-containing polymers—spatial resolution and electron density imaging

**Fig. 2:**
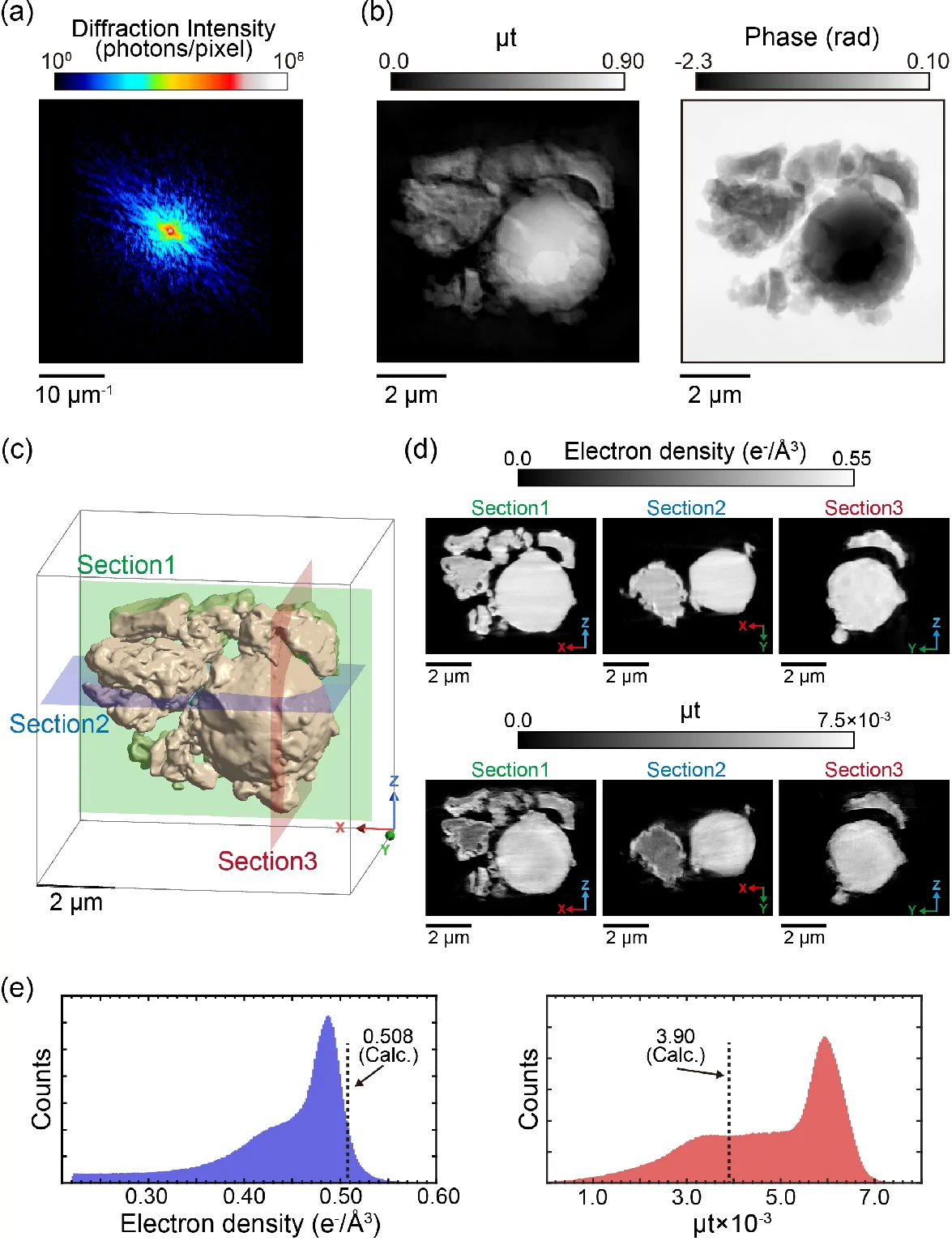
Ptychographic imaging and three-dimensional reconstruction of sulfurized poly(*n*-butyl methacrylate) (SPBMA) particles. (**a**) Representative far-field diffraction pattern recorded from an SPBMA particle. (**b**) Absorption (left) and phase (right) projection images reconstructed by ptychography at 2500 eV. (**c**) Isosurface rendering of the reconstructed three-dimensional electron-density volume, showing the overall particle morphology. (**d**) Cross-sectional views of (top) electron density and (bottom) absorption for Sections 1–3 in (**c**). (**e**) Histograms of (left) electron density and (right) absorption coefficient at 2500 eV within the particle interior. The mean ± standard deviation of the particle interior are 0.440 (± 0.067) e^−^/Å^3^ for electron density and 4.74 (± 1.48) × 10^–3^ for absorption at 2500 eV. The dotted lines indicate reference values estimated from bulk density, elemental composition determined by SEM–EDX, and the Henke tables. These values are in reasonable agreement with those estimated from bulk density and SEM–EDX measurements, considering the intrinsic heterogeneity of the sample and systematic uncertainties arising from calibration, reconstruction, and density estimation. This comparison demonstrates overall consistency with expected values while revealing internal compositional heterogeneity.

Figure 2 summarizes the reconstructed X-ray images of sulfur-containing polymer particles obtained through sulfur *K*-edge PXCT. Figure 2a presents a representative diffraction pattern of SPBMA, with scattering signals detected at spatial frequencies exceeding 10 µm^−1^. Figure 2b displays the absorption and phase images of the sample at 2500 eV, derived from the diffraction patterns, with a pixel size of 25 nm for the reconstructed sample projections. The ptychographic reconstruction may include a constant offset in both the phase and absorption (amplitude) images, as well as an indeterminacy in the linear phase ramp^36,37^. The images in Fig. 2b show the projections without the constant offsets in the phase and absorption, removed using a sample-free region on the membrane as a reference. No significant phase gradient was observed in the reconstructed phase image. Therefore, no explicit correction for the linear phase ramp was applied. Figure S4 exhibits an SEM image captured immediately after sample preparation, while Supplementary Note 4 outlines the imaging geometry employed for SEM imaging. The reconstructed particle exhibit spherical and non-spherical regions, consistent with SEM observations.

The phase volumetric data were adjusted to represent the electron density. The conversion to the electron density ${\rho}_\mathrm{ED}$ was performed using the relations:

1 $$\begin{aligned}  \rho_\mathrm{ED} &= -\frac{2\pi \delta }{{r_{e} \lambda^{2} }}, \hfill \\ \delta &= \frac{\lambda }{2\pi t}\Phi , \hfill \\ \end{aligned}$$

where *Φ* is the phase shift, ${r}_{e}$ is the classical electron radius, λ is the wavelength, *δ* is the real part of the complex refractive index, and *t* is the voxel edge length. Subsequently, particles and background regions were segmented from the electron density volume using Otsu’s method^38^. Figure 2c displays an isosurface rendering of the identified particles (also refer to Supplementary Movie 1). These particles had a maximum thickness of about 3.3 µm along the optical axis, illustrating the capability of the current approach to investigate micron-sized samples challenging to observe using electron microscopy. Figure 2d shows cross-sectional views of the three-dimensional reconstructed electron density and absorption images at 2500 eV in the X-Z, X-Y, and Y-Z planes. These sections correspond to Sections 1–3 in Fig. 2c. Due to the missing wedge associated with the limited angular range and the finite number of projections, stripe-like artifacts along the X direction and streak artifacts in the XY plane are observed in the sectional images. Although the structure along the optical axis (Y direction) is partially incomplete, the overall morphology of the sample is largely preserved owing to the angular coverage up to ± 84°. The spatial resolution of the volumetric images was assessed using Fourier shell correlation (FSC), a standard metric for isotropic resolution evaluation^39^. Figure S5a illustrates the FSC curves computed from the phase and absorption images. Supplementary Note 5 and Methods provide detailed FSC analyses and interpretation of the curves. By using the intersection of the FSC curve with the 1/2-bit threshold as the resolution criterion, resolutions of 63.1 nm and 69.6 nm were determined for the phase and absorption images, respectively. Owing to the silicon frame of the membrane blocking the scattering signal, projection images could not be obtained at tilt angles exceeding 84°, resulting in a missing wedge in the three-dimensional Fourier space. As Fourier-space information is constrained by the missing wedge, the resolution along the optical axis slightly degrades compared to directions perpendicular to the optical axis. Consequently, the resolution was separately evaluated for the in-plane and optical-axis directions. The in-plane resolution was determined from the intersection of the Fourier ring correlation (FRC) curve with the 1-bit threshold. The FRC curve was calculated from two independently reconstructed projection images at 2500 eV under normal incidence (0°). Figure S5b presents the FRC curves calculated from the phase and absorption projection images, revealing in-plane resolutions ${d}_{xz}$ of 68.2 nm for the phase images and 80.3 nm for the absorption images. Based on the relationship between in-plane and optical-axis resolutions (Method), the optical-axis resolution ${d}_{y}$ was estimated to be 73.3 nm for the phase images and 86.2 nm for the absorption images. Due to alignment imperfections of the projections with respect to the rotation axis, the three-dimensional resolution is expected to be lower than that of the two-dimensional images. The superior resolution obtained from FSC compared to FRC is likely due to noise suppression from the non-negative constraint applied during three-dimensional reconstruction. Overall, the achieved three-dimensional spatial resolution ranges from 70 to 80 nm.

Figure 2e illustrates histograms of the electron density and absorption μt within the particle. Regarding quantitative accuracy, incomplete back-projection from the missing wedge may lead to underestimated voxel values, while the non-negativity constraint may introduce a positive bias. Although the quantitative impact of these imperfections cannot be rigorously assessed, we used reference values for the electron density and absorption coefficient based on the bulk density, SEM–EDX composition, and the Henke tables^40^ (Supplementary Note 6). The estimated electron density (0.508 e⁻/Å^3^) is in reasonable agreement with the experimentally obtained median value (0.463 e⁻/Å^3^). Because the sample is heterogeneous, the values derived from averaged data should be regarded as approximate; nevertheless, the agreement supports the quantitative validity of the reconstruction. The median electron density of the particles was 0.463 e^−^/Å^3^, lower than that of typical light elements such as Ti-, Al-, and Si-based compounds^25^. Electron density imaging is thus well-suited for observing light-element materials, benefiting from strong phase contrast and large scattering cross sections in the tender X-ray regime. Additionally, the histograms did not exhibit simple Gaussian shapes, indicating compositional heterogeneity within individual particles and prompting further compositional and chemical-state analysis.

## Three-dimensional compositional analysis of sulfur-containing polymers

**Fig. 3:**
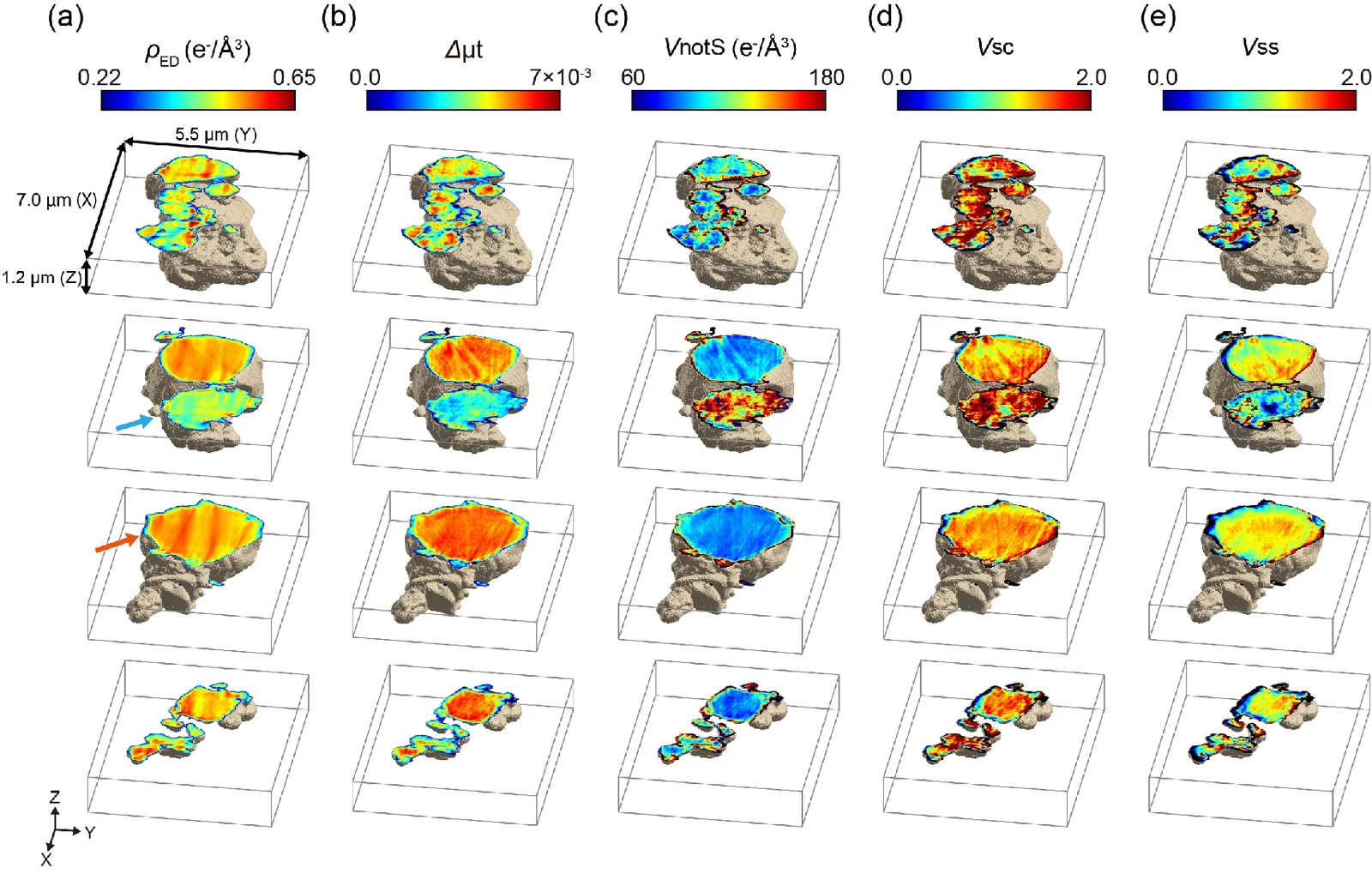
Cross-sectional parameter maps reflecting the composition of SPBMA. Representative cross-sectional slices from the same reconstructed particle are shown for (**a**) electron density ${\rho}_\mathrm{ED}$, (**b**) sulfur-related absorption contrast $\Delta \mu t$, (**c**) non-sulfur component parameter ${V}_\mathrm{notS}$, (**d**) S–C–bonded sulfur proxy ${V}_\mathrm{SC}$, and (**e**) S–S–bonded sulfur proxy ${V}_\mathrm{SS}$. Black pixels indicate negative Δμt in (**b**), Δμt values below the threshold in (**c**), and pixels with Δμt below the threshold or negative denominators in Eqs. 5 and 6 in (**d**, **e**). The Δμt threshold was set to Δμt *\ge* 8.19 × 10⁻^4^ based on a signal-to-noise ratio (SNR) criterion (SNR = Δμt/${\sigma}_\mathrm{full}\ge 3$ with ${\sigma}_\mathrm{full}$ = 2.73 × 10⁻^4^). The maps reveal pronounced compositional and chemical-state heterogeneity within the particle.

Three-dimensional compositional and chemical state heterogeneities in SPBMA were examined using absorption coefficients and electron density reconstructed at four energies near the sulfur *K*-edge. Figure 3a displays representative cross-sectional slices of the electron density ${\rho}_{\mathrm{ED}}$ alongside the particle morphology. The stripe-like patterns seen in the cross sections are attributed to reconstruction artifacts resulting from the limited angular range. The non-spherical regions, highlighted by blue arrows in Fig. 3a, exhibit lower electron density compared to the spherical regions marked by orange arrows. Given that SPBMA consists primarily of carbon, oxygen, and sulfur, the observed electron density contrast primarily stems from variations in sulfur concentration as the heaviest constituent element.

To analyze the sulfur distribution, the absorption difference $\Delta \mu t$ above and below the edge was calculated as:

2 $$\begin{aligned} \Delta \mu t &= \mu t_{\mathrm{post}} - \mu t_{\mathrm{pre}} \\ &  \approx 4\pi t\left\{ {\frac{{r_{e} \lambda_{{{\mathrm{post}}}} }}{2\pi }\mathop \sum \limits_{j} n_{j} \cdot f_{j}^{^{\prime\prime}} \left( {\lambda_{{{\mathrm{post}}}} } \right) - \frac{{r_{e} \lambda_{{{\mathrm{pre}}}} }}{2\pi }\mathop \sum \limits_{j} n_{j} \cdot f_{j}^{^{\prime\prime}} \left( {\lambda_{{{\mathrm{pre}}}} } \right)} \right\} \\ &  \approx 2r_{e} \lambda_{{{\mathrm{post}}}} t \cdot n_{{\mathrm{S}}} \cdot \left\{ {f_{{\mathrm{S}}}^{^{\prime\prime}} \left( {\lambda_{{{\mathrm{post}}}} } \right) - f_{{\mathrm{S}}}^{^{\prime\prime}} \left( {\lambda_{{{\mathrm{pre}}}} } \right)} \right\},\\ \end{aligned}$$

where ${\mu t}_{\mathrm{post}}$ and ${\mu t}_{\mathrm{pre}}$ are the absorption images at 2500 eV and 2463 eV, respectively; ${\lambda}_{\textrm{post}}$ and ${\lambda}_{\textrm{pre}}$ are the corresponding wavelengths; ${n}_{j}$ is the number density of element *j*; and ${f}_{j}^{{\prime}{\prime}}$ is the imaginary part of the atomic scattering factor ${f}_{j}$($={f}_{j}^{0}+{f}_{j}^{\prime}+{f}_{j}^{{\prime}{\prime}}$). The variation in atomic scattering factors of elements other than sulfur around the sulfur *K*-edge is negligible, and ${\lambda}_{\textrm{post}}\approx {\lambda}_{\textrm{pre}}$ in the present experiment. Under these assumptions, the relation indicates $\Delta \mu t$ is proportional to the product ${n}_\textrm{S}t$, which represents the line-integrated sulfur density within each voxel. Figure 3b illustrates a typical cross-section of $\Delta \mu t$, indicating higher sulfur levels in spherical areas and comparatively lower levels in non-spherical regions.

To further evaluate compositional variances, a parameter denoted as ${V}_{\textrm{notS}}$ was introduced, representing the electron density normalized by $\Delta \mu t$:

3 $$\begin{aligned} V_{{{\mathrm{notS}}}} &= \frac{{\rho_\mathrm{ED} }}{\Delta \mu t} \\& \approx \frac{{\mathop \sum \nolimits_{j} n_{j} \left\{ {Z_{j} + f_{j}^{\prime} \left( {\lambda_{{{\mathrm{post}}}} } \right)} \right\}}}{{2r_{e} \lambda_{{{\mathrm{post}}}} t \cdot n_{{\mathrm{S}}} \cdot \left\{ {f_{{\mathrm{S}}}^{^{\prime\prime}} \left( {\lambda_{{{\mathrm{post}}}} } \right) - f_{{\mathrm{S}}}^{^{\prime\prime}} \left( {\lambda_{{{\mathrm{pre}}}} } \right)} \right\}}} \\ & \propto Z_\mathrm{S} + \frac{{n_{{\mathrm{C}}} }}{{n_{{\textrm{S}}} }}Z_{{\mathrm{C}}} + \frac{{n_{{\mathrm{O}}} }}{{n_{{\mathrm{S}}} }}Z_\mathrm{O} , \\ \end{aligned}$$

where ${Z}_{j}$ is the atomic number of element *j*. The term ${f}_{j}^{0}\cong {Z}_{j}$ is assumed in the forward scattering limit. In addition, the following relation for δ was used:

4 $$\delta = \frac{{r_{e} \lambda^{2} }}{2\pi }\mathop \sum \limits_{j}^{{}} n_{j} \left( {Z_{j} + f_{j}^{\prime} } \right).$$

${V}_{\textrm{notS}}$ is a contrast parameter with unit of e^−^/Å^3^ that reflects variations in the electron density normalized by local sulfur concentration, highlighting regions enriched in low-Z elements (e.g., C and O). However, it does not directly represent the molar fraction or electron density of low-Z elements. Because ${V}_\textrm{notS}$ is defined as a ratio with $\Delta \mu t$ in the denominator, its stability is inherently sensitive to the magnitude of $\Delta \mu t$. In particular, when $\Delta \mu t$ approaches the noise level, small fluctuations in $\Delta \mu t$ can lead to large variations in ${V}_{\mathrm{n}\mathrm{o}\mathrm{t}\mathrm{S}}$, potentially introducing spurious contrast. In brief, because ${V}_\mathrm{notS}$ includes $\Delta \mu t$ in the denominator, it becomes sensitive to noise when $\Delta \mu t$ is small, leading to large fluctuations in ${V}_\mathrm{notS}$. To address this issue, the voxels used for evaluating ${V}_\mathrm{notS}$ were selected based on the noise level of $\Delta \mu t$. The standard deviation ${\sigma}_{\textrm{full}}$ of the random noise in the full dataset was derived from the difference between two independently reconstructed half datasets (see Supplementary Note 7). In this study, ${\sigma}_{\mathrm{full}}$ was estimated to be 2.73 × 10^–4^. Based on this, we defined a practical signal-to-noise ratio (SNR) as $\Delta \mu t/{\sigma}_\mathrm{full}$, and set a threshold of SNR *\ge* 3 ($\Delta \mu t$ *\ge* 8.19 × 10^–4^). This SNR-based criterion was found to be more restrictive than the threshold of $\Delta \mu t$ *\ge* 2.10 × 10^–4^ derived from Tukey’s fences (Supplementary Note 8). Figure 3c displays another cross-section of ${V}_{\textrm{notS}}$. The parameter mapping reveals elevated values in regions with reduced sulfur content. Based on the elemental makeup, regions with high ${V}_\mathrm{notS}$ exhibit enrichment in carbon and oxygen.

A previous report indicated that sulfur in SPBMA primarily exists in S–C and S–S bonding configurations, with a minor contribution from S=C bonding species^27^. This study focuses on evaluating the spatial distribution of sulfur associated with the dominant S–C and S–S bonds. Owing to the complex absorption spectrum of SPBMA, a simple linear combination of pure S–C and S–S reference spectra cannot represent it, making the unambiguous decomposition of chemical phases challenging. To characterize sulfur chemical states, two dimensionless metrics, ${V}_{\mathrm{SC}}$ and ${V}_{\mathrm{SS}}$, were defined as follows:

5 $$V_{{{\mathrm{SC}}}} = \frac{{\mu t_{{{\mathrm{SC}}}} - \mu t_{{{\mathrm{post}}}} }}{\Delta \mu t},$$

6 $$V_{{{\mathrm{SS}}}} = \frac{{\mu t_{{{\mathrm{SS}}}} - \mu t_{{{\mathrm{post}}}} }}{\Delta \mu t},$$

where ${\mu t}_{\textrm{SC}}$ and ${\mu t}_{\textrm{SS}}$ are the absorption images at 2469.6 eV and 2470.7 eV, respectively. These parameters represent the local spectral contrast at the S–C and S–S energies relative to a post-edge reference, normalized by $\Delta \mu t$. Although the voxel values and sensitivity depend on the selected energies, the use of energies near the respective peaks allows ${V}_{\textrm{SC}}$ and ${V}_{\textrm{SS}}$ to be interpreted as spectral proxies that are approximately proportional to the local abundance of sulfur in each bonding environment under a linear-response approximation. Because the SPBMA spectrum is complex and the peaks overlap, each parameter also contains contributions from multiple sulfur species. They do not directly represent the exact molar ratio of sulfur in S–C and S–S bonding environments. Instead, they should be interpreted as relative indicators of spatial enrichment or depletion in S–C and S–S bonding environments. Because the SPBMA spectrum is complex and peaks overlap, each parameter includes contributions from multiple sulfur species. Figures 3d and 3e show three-dimensional cross-sections of ${V}_{\textrm{SC}}$ and ${V}_{\textrm{SS}}$, revealing heterogeneous spatial distributions. The distribution of ${V}_\textrm{SC}$ exhibited relatively uniform values within spherical particles but tended to be slightly higher in non-spherical particles. In addition, ${V}_\mathrm{SS}$ exhibited relatively high values high throughout spherical regions and slightly lower values in non-spherical regions. These findings indicate that sulfur in S–S bonding configurations is preferentially concentrated within spherical regions, whereas non-spherical regions contain significantly lower fractions of S–S-bonded sulfur. In contrast, ${V}_\mathrm{SC}$ consistently shows high values across the entire particle, suggesting a more uniform distribution of sulfur involved in S–C bonding. Detailed derivations of the parameters in Eqs. (1–6) are provided in Supplementary Note 9. Three-dimensional maps of the five parameters (${\rho}_\mathrm{ED}$, $\Delta \mu t$, ${V}_{\mathrm{notS}}$, ${V}_{\mathrm{SC}}$, and ${V}_{\mathrm{SS}}$) are also available in Supplementary Movies 2–6. Similar artifacts to those observed in Fig. 2d are also present in the supplementary movies 2–5; however, these are likely not intrinsic to the sample but originate from measurement or reconstruction processes.

**Fig. 4:**
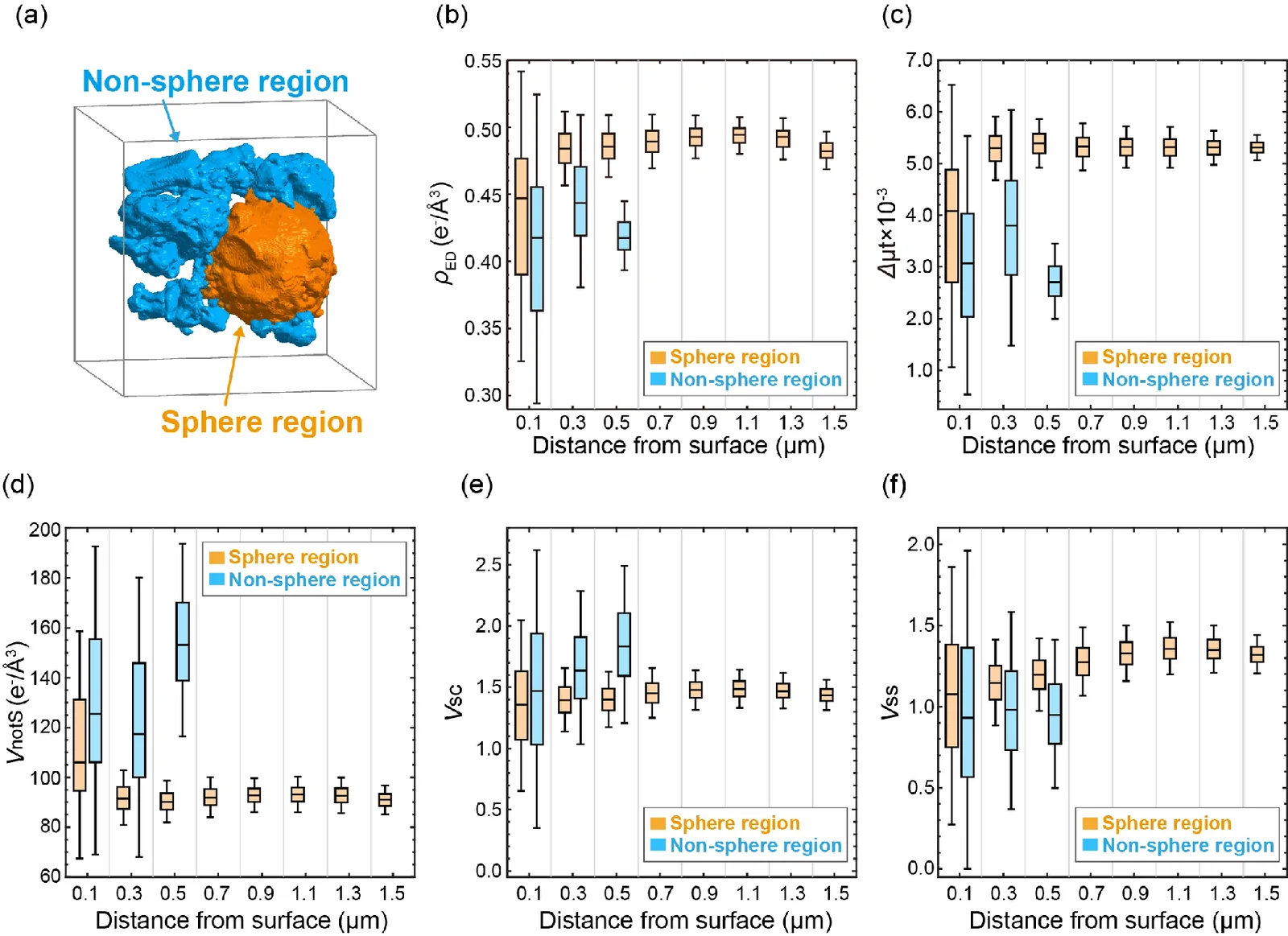
Distance-dependent compositional analysis of spherical and non-spherical regions in an SPBMA particle. (**a**) Segmentation of a representative SPBMA particle into spherical and non-spherical regions based on particle morphology. Distance-dependent distributions of (**b**) electron density (${\rho}_\mathrm{ED}$), (**c**) sulfur-related absorption contrast ($\Delta \mu t$), (**d**) non-sulfur contrast parameter (${V}_\mathrm{notS}$), (**e**) S–C–bonded sulfur proxy (${V}_\mathrm{SC}$), and (**f**) S–S–bonded sulfur proxy (${V}_\mathrm{SS}$) are shown separately for the spherical and non-spherical regions. In panels (**b**–**f**), voxels were grouped into bins of 200 nm width based on their distance from the particle surface, computed using a Euclidean distance transform. Bin centers are shown on the horizontal axis. Box-and-whisker plots indicate the median (central line), interquartile range (box), and whiskers extending to 0.75 × IQR from the box edges. Voxels with negative *Δ*μt in (**c**), *Δ*μt values below the threshold in (**d**), and *Δ*μt values below the threshold or negative denominators in Eqs. 5 and 6 in (**e**, **f**) were excluded from the statistical analysis for the box-and-whisker plots. These results reveal that spherical regions are relatively sulfur-rich and enriched in S–S bonding, whereas non-spherical regions are relatively enriched in low-Z elements, such as carbon and oxygen, with a higher contribution from S–C–bonded sulfur.

Comparison of spherical and non-spherical regions revealed differences in parameter distributions. To assess these variances, the particle volume data were initially divided into spherical and non-spherical regions (Supplementary Note 10)^41,42^. Figure 4a illustrates the segmentation outcome. Subsequently, the volumetric data were categorized into 200 nm intervals based on the distance from the particle surface. The distance from the particle surface was computed using a Euclidean distance transform^41^. Within each distance bin, parameter distributions were separately analyzed for spherical and non-spherical regions. Figure 4b–f summarize the distance-dependent distributions of both regions using box-and-whisker plots. The box represents the interquartile range, with the median denoted by the central line, while the whiskers indicate data variability. Figure 4b demonstrates that the median electron density is lower in non-spherical regions compared to spherical regions. Correspondingly, non-spherical regions display lower values and a higher proportion of non-sulfur components in Figs. 4c and 4d. These findings suggest that spherical regions are sulfur-rich, whereas non-spherical regions are relatively enriched in carbon and oxygen. The box plots in Fig. 4c also indicate that the sulfur concentration was relatively higher in the bulk region (*\ge* 200 nm) than on the near-surface region (*łe* 200 nm) of the spherical particles. Comparison of the median ${V}_{\mathrm{SC}}$ and ${V}_{\mathrm{SS}}$ values in Figs. 4e and 4f reveals a reduced abundance of S–C–bonded sulfur and an enrichment of S–S–bonded sulfur in spherical regions. Together, these analyses unveil distinct three-dimensional correlations among particle morphology, elemental composition, and sulfur bonding states within individual SPBMA particles.

## Chemical states in projection obtained via 2D X-ray spectroscopic ptychography

**Fig. 5:**
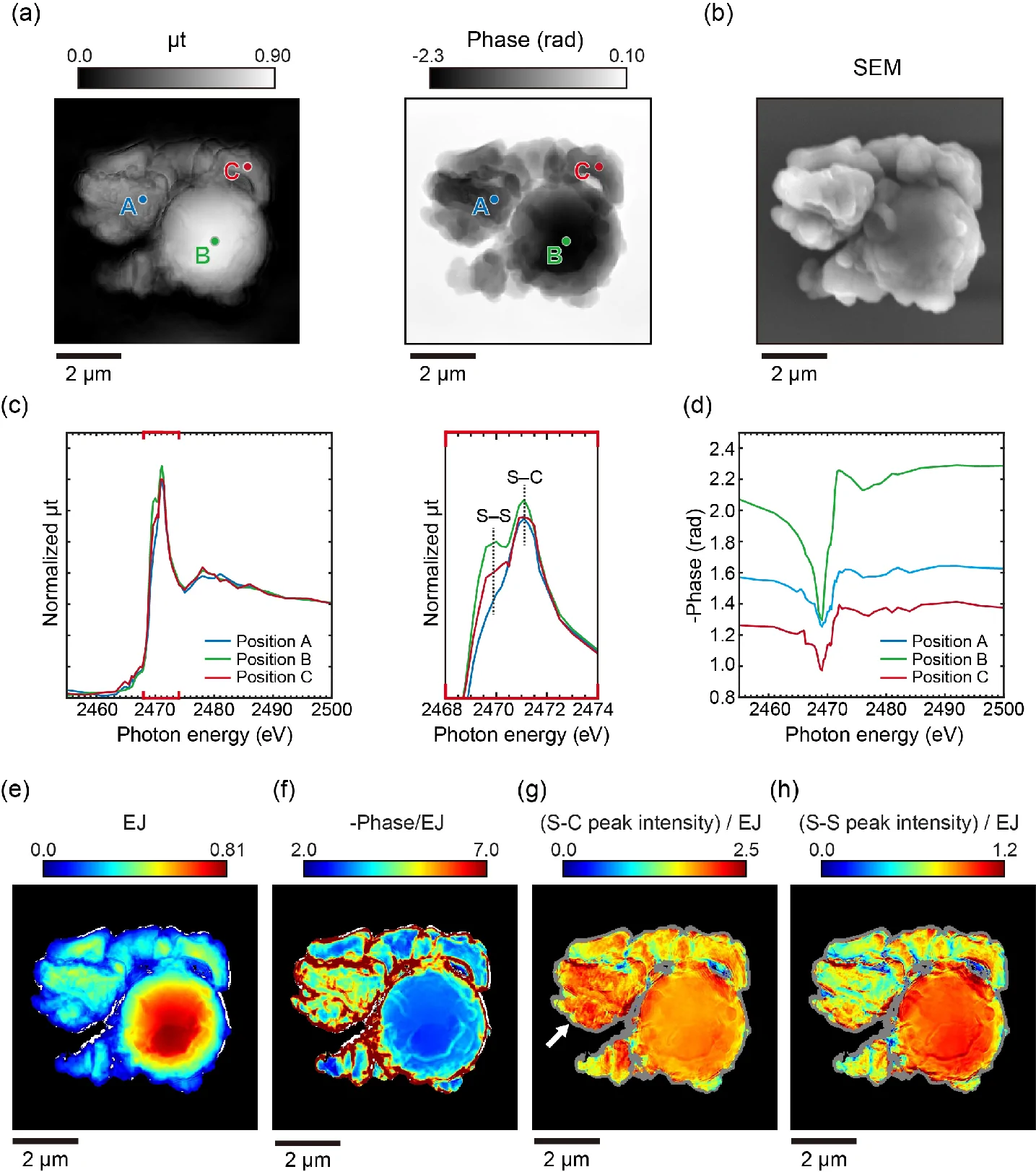
Validation of three-dimensional PXCT results by spectroscopic ptychography. (**a**) Absorption (left) and phase (right) projection images of SPBMA at 2500 eV acquired after four PXCT scans. (**b**) SEM image acquired after the synchrotron radiation experiment, showing the particle morphology after X-ray exposure. (**c**) Spatially resolved sulfur *K*-edge X-ray absorption spectra extracted from representative regions within the particle, showing the full energy range (left) and the white-line region (right). (**d**) Spatially resolved sulfur *K*-edge phase spectra extracted from the same regions. (**e**) Edge-jump (EJ) map derived from energy-averaged absorption images. (**f**) EJ-normalized phase shift map. (**g**) EJ-normalized S–C peak intensity map. (**h**) EJ-normalized S–S peak intensity map. In panels (**e**) and (**f**), pixels with negative EJ values are shown in white. In panels (**g**) and (**h**), pixels with EJ < 0.10, where the white-line features are not well defined, are shown in gray. Panels (**e**)–(**h**) represent two-dimensional projection analogs of $\Delta \mu t$, ${V}_\textrm{notS}$, ${V}_\mathrm{SC}$, and ${V}_\mathrm{SS}$, respectively. The spectroscopic ptychography results are consistent with the three-dimensional chemical-state distributions obtained by PXCT.

To validate the three-dimensional chemical-state information acquired through PXCT, X-ray spectroscopic ptychography was conducted on the same sample post PXCT measurements. Diffraction patterns were captured at 46 energy points spanning 2455–2500 eV around the sulfur *K*-edge. Figure 5a illustrates the phase and absorption images of the sample at 2500 eV. Figure 5b provides an SEM image post synchrotron experiments. X-ray absorption and phase spectra were derived from the image stacks to explore the chemical state. Figures 5c and 5d exhibit the spatially resolved sulfur *K*-edge X-ray absorption spectra. These absorption spectra are in good agreement with previously reported SPBMA spectra^27^ and the CEY spectrum (Fig. S1). The phase spectra represent contributions from both the projected electron density and the dispersion effects of absorbing elements. Because phase images generally exhibit a higher signal-to-noise ratio than absorption images, phase spectra have also been explored for chemical-state analysis^18^. A decrease in phase shift near the absorption edge is attributed to sulfur dispersion, in line with prior studies. Within the energy range investigated in this study, the phase image at 2500 eV is approximately proportional to the projected electron density. Relative to the phase image captured pre-PXCT, the post-PXCT phase image reveals a noticeable expansion in particle morphology.

To analyze the morphological changes, the edge-jump (EJ) was initially determined by comparing absorption images below and above the edge. Figure 5e illustrates the EJ map of the specimen, highlighting the spatial distribution of sulfur-related contrast. Since sulfur dominates the absorption above the sulfur *K*-edge, the absorption image in Fig. 2b primarily reflects contrast associated with sulfur. The resemblance between the EJ map and the absorption image pre-PXCT indicates the preservation of sulfur’s spatial distribution. For assessing elements other than sulfur, Fig. 5f presents the phase shift normalized by the EJ. This normalization diminishes sulfur-related contrast while amplifying non-sulfur element contrast. It also compensates for thickness variations, accentuating bulk components internally and surface components externally in the particle. The surface area in Fig. 5f displays higher contrast than the particle interior, suggesting an abundance of non-sulfur elements on the surface. As the surface phase shift is not excessively large, the surface density is deemed similar to that of the polymer. While X-ray-induced alterations to the sample cannot be ruled out, sulfur distribution and absorption spectra were predominantly conserved. These findings imply that the observed morphological alterations likely stem from surface contamination rather than bulk chemical modifications. Enhancing the vacuum level of the sample chamber, currently around ~ 10⁻^1^ Pa, could mitigate such contamination.

Finally, the chemical state maps were assessed using absorption images obtained near the S–S and S–C bonding peaks. The intensity peak for each bond was standardized by the EJ. Figures 5g and 5h display the EJ-normalized S–C and S–S peak intensities, respectively. These maps indicate values approximately proportional to the local abundance of sulfur engaged in S–C or S–S bonding. Sulfur involved in S–C bonding is evenly dispersed throughout the particle, while S–S-bonded sulfur exhibits a spatially non-uniform distribution. The non-spherical region indicated by the white arrow in Fig. 5g suggests a relative enrichment of S–C-bonded sulfur compared to the surrounding regions, whereas the spherical region displays a relative enrichment of S–S-bonded sulfur in Fig. 5h. These spatial trends observed in the EJ-normalized S–C and S–S peak intensity maps are consistent with those in the three-dimensional ${V}_\textrm{SC}$ and ${V}_\textrm{SS}$ maps obtained by PXCT analysis in Figs. 3d and 3e. Additionally, the spherical region’s tendency to be enriched in S–S-bonded sulfur, contrasted with the relatively lower content in the non-spherical region, was further validated by spectroscopic ptychography of other particles (Supplementary Note 11). These results provide independent experimental verification of the three-dimensional sulfur chemical state distributions uncovered by PXCT.

## Discussion

In this study, we demonstrated that spectroscopic PXCT at the sulfur *K*-edge enables three-dimensional visualization of electron density, sulfur distribution, and sulfur chemical states in sulfur-containing polymers several micrometers in thickness. The consistency between the PXCT results and the independent two-dimensional spectroscopic ptychography measurements confirms that the observed chemical-state heterogeneity is intrinsic to the material rather than a reconstruction artifact. Although higher chemical specificity would require more energy points, reference compounds, and advanced spectral unmixing, the PXCT reconstructions at the selected four energies showed trends consistent with the projection data. The EJ map can be regarded as the projection-space counterpart of $\Delta \mu t$ in PXCT. The EJ-normalized phase shift is related to the projection analogue of ${V}_\textrm{notS}$, while the EJ-normalized S–S and S–C peak intensities are related to those of ${V}_\textrm{SS}$ and ${V}_\textrm{SC}$, respectively. Achieving high spatial resolution at the sulfur *K*-edge is challenging because the available photon flux is significantly reduced by X-ray attenuation in air, window materials, and sample supports, as well as by the limited efficiency of focusing optics. Moreover, conventional PXCT setups using pinholes or zone plates necessitate an aperture placed immediately upstream of the sample, which limits the sample size and experimental geometry. In contrast, the total-reflection mirror–based focusing optics utilized in this work offer high focusing efficiency at the sulfur *K*-edge while maintaining a long working distance. Consequently, this optical configuration enabled sufficient photon flux for high-resolution PXCT while considerably relaxing constraints on sample size, shape, and surrounding environment. This advantage facilitates high-resolution three-dimensional imaging of nanoscale-to-microscale regions in physically supported samples such as particles, thin films, and active regions of operating battery devices.

PXCT provides high-resolution, three-dimensional imaging from the near-surface to deep within the bulk of micrometer-sized specimens, simultaneously exploiting both absorption and electron-density contrast in a transmission geometry. In contrast, conventional scanning X-ray microscopy does not directly provide electron-density sensitivity, limiting its ability to resolve fine internal structures at the nanoscale. In this study, five spatially resolved compositional parameters were quantitatively extracted from only four X-ray energy points, highlighting the high information content of the approach. From PXCT, the sulfur concentration in spherical particles differed between the near-surface and bulk regions, whereas in non-spherical regions S–C–bonded sulfur exhibited a monotonic increase with depth, while no clear trends were observed for the other parameters. Spectroscopic ptychography measurements integrate information along the beam direction and therefore cannot distinguish overlapping structures in depth with complex geometries. Nevertheless, the consistent enrichment patterns observed in both the 2D spectroscopic maps and the 3D PXCT volume support the interpretation that the observed heterogeneity is intrinsic rather than a PXCT artifact. PXCT overcomes these limitations by directly resolving the three-dimensional distribution of chemical states, providing nanoscale insights into reaction fronts and interfacial phenomena in advanced functional materials.

Despite these advances, several challenges persist for the current approach. At the sulfur *K*-edge, the applicable sample thickness is practically limited to a few micrometers (typically up to ~ 3–5 µm for low-Z matrices without significant concentrations of heavier elements), due to the combined effects of absorption and limited penetration depth. For more strongly absorbing or multi-component systems, trade-offs between spatial resolution, field of view, and photon energy selection are required. In particular, the use of higher photon energies can relax absorption constraints, albeit at the cost of reduced chemical sensitivity. Irradiation with tender X-rays close to the soft X-ray regime (< 2000 eV) may induce radiation damage to the sample. Additionally, the extended measurement time needed for CT acquisition presents practical limitations on experimental conditions. Future advancements, such as enhanced low-dose imaging techniques, cryogenic measurements, and on-the-fly scanning capabilities, are anticipated to address these constraints and broaden the scope of the current approach^43–46^.

In conclusion, the current study shows that, even at the highly absorbing sulfur *K*-edge, three-dimensional analysis of both light-element materials and sulfur bonding states is achievable with a suitable measurement system. This research introduces a novel experimental platform for spatially resolving the structure–function relationships of light-element-based materials. The method is anticipated to have wide-ranging applications in the three-dimensional structural and chemical-state analysis of functional polymers, energy-related materials, and other light-element-based systems.

## Methods

### Sample preparation

Spherical poly(*n*-butyl methacrylate) particles with an average particle size of 5 μm (25 wt%, Shimizu Chemical Industries Co., Ltd.) and sulfur (75 wt%, Kishida Chemical Co., Ltd.) were combined and heated at 613 K for 2 h in a small muffle furnace under a continuous argon flow. Subsequently, excess sulfur was eliminated by vacuum heating at 563 K for 3 h using a rotary pump. The density of the sample after desulfurization was 1.841 g/cm^3^. The average composition and chemical state of the samples were analyzed using XAS in CEY mode and scanning electron microscopy with SEM–EDX (Supplementary Note 1 and 2). The resulting powder was dispersed in ethanol and then deposited onto a 200 nm-thick SiN membrane (NX10500D, Norcada).

### Ptychographic X-ray computed tomography

Ptychographic measurements were conducted at the BL10U beamline of NanoTerasu using AKB mirror focusing optics^29^. Synchrotron radiation emitted from a vacuum-sealed undulator was monochromatized using a three-mirror higher-harmonic rejection system, followed by a Si(111) double-crystal monochromator. The rejection mirrors were coated with carbon, nickel, and carbon, and operated at grazing incidence angles of 5, 10, and 5 mrad, respectively. After monochromatization, the X-rays were shaped by a downstream slit to 10 µm (H) × 100 µm (V) and subsequently focused in two dimensions using a pair of AKB mirrors housed in the mirror chamber. The focal spot size was approximately 1.48 µm (H) × 1.28 µm (V), as estimated from the reconstructed probe intensity distribution. Compared with a previously reported setup, the ion chamber was removed. The mirror chamber was directly connected to the sample chamber and separated by a 37.5 µm-thick Kapton film, which significantly reduced attenuation of the incident X-ray flux. The photon flux at the sample position reached approximately 1 × 10⁹ photons s⁻^1^ at 2500 eV. The sample was positioned at the focal plane of the optics. Ptychographic scanning was performed using piezoelectric stages (PI GmbH, P-621.ZLC and P-621.1CL). Diffraction intensity patterns were recorded with a CITIUS 840 k detector positioned approximately 1.87 m downstream of the sample. Prior to PXCT measurements, the incident probe wavefield was characterized by ptychographic measurements of a 200 nm-thick Ta test pattern (NTT-AT Corp.). Probe and object functions were reconstructed using the extended ptychographic iterative engine (ePIE) algorithm^33,47^. All data analyses in this study were performed using MATLAB (R2025a, MathWorks Inc., Natick, MA, USA). The reconstructed probe function and the phase image of the test pattern are shown in Supplementary Note 3. For PXCT measurements, sample rotation was achieved using a rotation stage (Xeryon, XRT-U-30) mounted on the piezo stage. The rotation axis was aligned before measurements using two translational stages (Xeryon, XLS-1-30). The reference hole was tracked at multiple projection angles to determine and correct the center of rotation of the sample. After alignment, overlapping ptychographic scans were acquired with a scan step of 500 nm over a grid of 16 × 14 positions, using an exposure time of 0.5 s per point. Ptychographic measurements were collected over an angular range of ± 84° with a step size of 1.5°, yielding 113 projection images per energy. Each ptychographic scan required approximately 4 min. PXCT measurements were performed at four X-ray energies near the sulfur *K*-edge: 2500 eV (above the absorption edge), 2463 eV (below the absorption edge), 2470.7 eV (near the S–S bonding peak), and 2469.6 eV (near the S–C bonding peak). Each CT dataset required approximately 8 h, and measurements were conducted sequentially in the order listed.

### Image reconstruction

Reconstruction of the SPBMA projection images was performed using the refractive index-based ptychographic iterative engine algorithm with mixed-state and position correction^32–34^. The reconstructed object function $\widetilde{O}$, which depicts the projected complex refractive index of the sample, is expressed as:

7 $$\begin{array}{c}\tilde{O} = \frac{2\pi }{\lambda }\left( { - \smallint \delta dy} \right) + \frac{2\pi i}{\lambda }\left( {\smallint \beta dy} \right) .\end{array}$$

Here, the complex refractive index was defined as $n=1-\delta +i\beta$, and the X-ray wavelength as *λ*. The phase and absorption images were obtained from the real part and twice the imaginary part of the complex object function, respectively. For the reconstruction, the initial probe function was the wavefield reconstructed using the Ta test chart (Supplementary Note 3). The initial object function was set to a uniform value with *δ =0* and *β =0*. A total of 800 iterations were performed. In the first 300 iterations, the real part of the object function guided the imaginary part to enhance convergence^48^. After 100 iterations, phase unwrapping based on phase differences between adjacent pixels was applied to the real part. The pixel size of the reconstructed images was 25 nm. The reconstructed object functions were converted into phase and absorption images and stacked along the angular and energy dimensions. For each energy, angular stacks were aligned sequentially using cross-correlation, center-of-mass alignment, and a fast projection algorithm^36,49,50^. The alignment shifts were determined from the phase images, and the absorption images were shifted by the same pixel offsets. After alignment, three-dimensional reconstruction was performed using the simultaneous iterative reconstruction technique with a non-negativity constraint for 300 iterations^35^. The radiation dose delivered during the CT measurements was estimated to be $5.18\times {10}^{9}$ Gy (Supplementary Note 12)^51^.

### Assessment of spatial resolution

The isotropic spatial resolution of the three-dimensional reconstructed data was assessed through Fourier shell correlation (FSC), defined as follows^39^:

8 $$\begin{array}{c}\mathrm{FSC}(q) = \frac{{\sum_{\mathrm{shell}} F_{1}(q)\,F_{2}^{*}(q)}}{{\sqrt {\sum_{\mathrm{shell}} \left |F_{1}(q) \right|^{2} \sum_{\mathrm{shell}} \left| F_{2}(q) \right|^{2} } }} .\end{array}$$

Here, *q* denotes the spatial frequency, and ${F}_{1}$ and ${F}_{2}$ are the complex Fourier amplitudes of the three-dimensional images reconstructed from two statistically independent projection datasets obtained by interleaving the projection angles. The asterisk (*) denotes the complex conjugate, and ${\Sigma}_\textrm{shell}$ represents summation over a spherical shell in Fourier space corresponding to the spatial frequency *q*. The 113 projection images were sorted by projection angle and divided into two datasets by alternately assigning the images. These two datasets were utilized to compute the FSC curve. The isotropic spatial resolution was defined as the reciprocal of the spatial frequency at which the FSC curve intersected the 1/2-bit threshold criterion.

The in-plane spatial resolution ${d}_{xz}$ was evaluated using the Fourier ring correlation (FRC) as defined by the following Equation^39^:

9 $$\begin{array}{c} \mathrm{FRC}\left( q \right) = \frac{{\mathop \sum \nolimits_{\mathrm{ring}} F_{\mathrm{proj},1}(q)\,F_{\mathrm{proj},2}^{*} (q)}}{{\sqrt {\mathop \sum \nolimits_{\mathrm{ring}} \left| {F_{\mathrm{proj},1}(q)} \right|^{2} \, \mathop \sum \nolimits_{\mathrm{ring}} \left| {F_{\mathrm{proj},2} (q)} \right|^{2} } }} . \end{array}$$

Here, $F_{\mathrm{proj},1}$ and $F_{\mathrm{proj},2}$ denote complex amplitudes in Fourier space reconstructed from two independent projection datasets. Σ_ring_ represents summation over Fourier-space pixels on the ring at spatial frequency *q.* At 2500 eV, two independent ptychographic measurements at a projection angle of 0° were performed twice under identical conditions, as described in the PXCT section, prior to PXCT. The reconstructed projection images were used to calculate the FRC. The in-plane spatial resolution was defined as the inverse of the spatial frequency at which the FRC curve intersected the 1-bit threshold criterion. For limited-angle CT, the spatial resolution along the optical axis, ${d}_{y}$, was calculated from the maximum tilt angle *α* and the in-plane resolution ${d}_{xz}$ using the following relation^52^:

10 $$\begin{array}{c} d_{y} = d_{xz} \, \sqrt {\frac{\alpha + \sin \alpha \cos \alpha }{{\alpha - \sin \alpha \cos \alpha }}}. \end{array}$$

### Compositional parameters in three-dimensional space

For compositional analysis, the three-dimensional phase image was converted to the refractive index components *δ*. The electron density, ${\rho}_\mathrm{ED}$, was subsequently determined from *δ* at 2500 eV using Eq. (1). As a parameter proportional to the line-integrated sulfur density within each voxel, $\Delta \mu t$ was calculated from the difference between absorption images at 2500 eV and 2463 eV using Eq. (2). Subsequently, ${V}_{\mathrm{notS}}$ was defined as the ratio of ${\rho}_\mathrm{ED}$ to *μ t*. To mitigate the effect of noise in voxels with small $\Delta \mu t$, an SNR-based voxel selection was applied. The noise level was estimated using a split-data approach (see Supplementary Note 7 for details), where the measured data were divided into two subsets and independently reconstructed using the same procedure as described above. The difference between the two $\Delta \mu t$ volumes was used to estimate the noise variance, assuming independent noise contributions with comparable statistical properties. The noise standard deviation in the full dataset, ${\sigma}_\mathrm{full}$, was approximated. The signal to noise ratio was defined as SNR = $\mathit{\Delta} \mu t$/${\sigma}_\mathrm{full}$, and voxels with SNR ≥ 3 (corresponding to $\mathit{\Delta} \mu t$ ≥ 8.19 × 10⁻^4^) were selected for subsequent ${V}_\mathrm{notS}$ analysis. This threshold was more restrictive than the Tukey’s fence (Supplementary Note 8)^53^. Subsequently, two dimensionless metrics, ${V}_\mathrm{SC}$ and ${V}_\mathrm{SS}$, were defined as the differences between the absorption images near the S–C (2469.6 eV) and S–S (2470.7 eV) peaks and the post-edge reference image at 2500 eV, normalized by $\Delta \mu t$. For these two parameters, voxels with SNR < 3 and those with negative numerator values in Eqs. (5 and 6) were excluded from the analysis.

### X-ray spectroscopic ptychography

After four PXCT measurements, X-ray spectroscopic ptychography was conducted on the same SPBMA particles. Using a slit size of 10 µm (H) × 100 µm (V), diffraction patterns were gathered at 46 energy points ranging from 2455 to 2500 eV near the sulfur *K*-edge. For each energy point, the sample underwent raster scanning in a 16 × 16 grid with a 500 nm step size and an exposure time of 0.5 s per point. Additional measurements were carried out in four distinct fields of view containing different particles from the same batch. In this scenario, diffraction patterns were captured at 30 energy points spanning from 2455 to 2500 eV. The scanning step size remained at 500 nm, and the exposure time was fixed at 0.5 s per point.

Phase retrieval was conducted on all collected diffraction patterns to reconstruct the sample images using the ePIE algorithm with mixed states and position correction, totaling 800 iterations^33,34,47^. The initial probe function utilized was the wavefront obtained from the test chart measurements. The initial object was defined with a uniform amplitude of unity and zero phase. To expedite convergence, the phase image guided the absorption (amplitude) image during the initial 300 iterations^48^. Kramers–Kronig-based real-space constraints^54,55^ were not applied in this study, as sufficient contrast and consistent spectral features were obtained by independently reconstructing the real and imaginary parts of the refractive index under the present experimental conditions. The pixel size for all reconstructed images was standardized to 25 nm.

### Analysis of spectroscopic data using projection images

Spectroscopic ptychography data were utilized to quantitatively assess composition-related parameters and chemical-state distributions of the sample. Chemical-state maps were derived from energy-averaged absorption images obtained below and above the absorption edge, as well as near the S–S and S–C bonding peaks. The averaged images are designated as $\langle \mu t_\mathrm{post}\rangle_E$ for energies above the absorption edge, ${\langle \mu {t}_\mathrm{pre}\rangle }_{E}$ for energies below the edge, ${\langle \mu {t}_\mathrm{SS}\rangle }_{E}$ for energies near the S–S bonding peak, and ${\langle \mu {t}_\mathrm{SC}\rangle }_{E}$ for energies near the S–C bonding peak. Energy averaging encompassed 2492.0–2500.0 eV for ${\langle \mu {t}_\mathrm{post}\rangle }_{E}$, 2455.0–2463.3 eV for ${\langle \mu {t}_\mathrm{pre}\rangle }_{E}$, 2469.3–2470.2 eV for ${\langle \mu {t}_\mathrm{SS}\rangle }_{E}$, and 2470.9–2471.3 eV for ${\langle \mu {t}_\mathrm{SC}\rangle }_{E}.$ Subsequently, utilizing these averaged images, four parameters reflecting the sulfur content, chemical state, and composition of the sample were calculated as detailed below:

11 $$\begin{array}{c}{\mathrm{EJ} = \langle \mu t_{\mathrm{post}}\rangle_E - \langle \mu t_{\mathrm{pre}}\rangle_E},\end{array}$$

12 $$\begin{array}{c}\frac{-\mathrm{Phase}}{\mathrm{EJ}}= \frac{-\mathrm{Phase}}{\langle \mu t_{\mathrm{post}}\rangle_E - \langle \mu t_{\mathrm{pre}}\rangle_E},\end{array}$$

13 $$\begin{array}{c}\frac{\mathrm{S\mathrm{-}S\ peak\ intensity}}{\mathrm{EJ}}=\frac{\langle\mu t_{\mathrm{SS}}\rangle_E - \langle \mu t_{\mathrm{post}} \rangle_E}{\langle \mu t_{\mathrm{post}} \rangle_E - \langle \mu t_{\mathrm{pre}} \rangle_E},\end{array}$$

14 $$\begin{array}{c}\frac{\mathrm{S\mathrm{-}C\ peak\ intensity}}{\mathrm{EJ}}=\frac{\langle\mu t_{\mathrm{SC}}\rangle_E - \langle \mu t_{\mathrm{post}} \rangle_E}{\langle \mu t_{\mathrm{post}} \rangle_E - \langle \mu t_{\mathrm{pre}} \rangle_E}. \end{array}$$

Here, phase represents the phase image at 2500 eV. Segmentation of particles and background regions was performed by applying *k*-means clustering to this phase image^56^. The EJ-normalized phase shift was determined solely for pixels with EJ greater than zero. The EJ-normalized intensities of the S–S and S–C peaks were assessed using pixels with EJ greater than or equal to 0.10, where the white-line peak characteristics were clearly defined.

## Supplementary Information

Below is the link to the electronic supplementary material.

Supplementary Material 1

Supplementary Material 2

Supplementary Material 3

Supplementary Material 4

Supplementary Material 5

Supplementary Material 6

Supplementary Material 7

## Data Availability

The datasets generated and/or analysed during the current study are available from the corresponding author upon reasonable request due to their large size.

## Code availability

No custom code was developed that is central to the conclusions of this study.
