# Supplementary material for: Three-dimensional imaging of sulfur chemical states in polymers with micrometer thickness using sulfur K-edge ptychographic tomography
Source: Sci Rep. 2026 May 16;16:26655. doi: 10.1038/s41598-026-52630-4 (PMC13507342; doi:10.1038/s41598-026-52630-4)
Supplement: Supplementary file 1 [file 41598_2026_52630_MOESM7_ESM.docx]

Supplemental Information for

**Three-dimensional imaging of sulfur chemical states in polymers with micrometer thickness using sulfur *K*-edge ptychographic tomography**

Yuhei Sasaki^1,2,3*^, Nozomu Ishiguro^1,2^, Masaki Abe^1,2,4^, Shuntaro Takazawa^1,2^, Naru Okawa^1,2,3^, Mihiro Ikenaga^1,2,3^, Fusae Kaneko^1,5^, Yukio Takahashi^1,2,4,6*^

^1^ International Center for Synchrotron Radiation Innovation Smart (SRIS), Tohoku University, 468-1 Aramaki Aza-Aoba, Aoba-ku, Sendai, 980-8572, Japan;

^2^ RIKEN SPring-8 Center, 1-1-1 Kouto, Sayo-cho, Sayo-gun, Hyogo 679-5148, Japan;

^3^ Department of Metallurgy, Graduate School of Engineering, Tohoku University, 6-6-2 Aoba-yama, Aoba-ku, Sendai 980-8579, Japan;

^4^ Institute of Multidisciplinary Research for Advanced Materials (IMRAM), Tohoku University, 2-1-1 Katahira, Aoba-ku, Sendai 980-8577, Japan;

^5^ Sumitomo Rubber Industries, Ltd., 2-1-1 Tsutsui, Chuo, Kobe, Hyogo 651-0071, Japan;

^6^ Institute for Materials Research, Tohoku University, 2-1-1 Katahira, Aoba-ku, Sendai, 980-8577, Japan;

* Corresponding author: yuhei.sasaki.s6@dc.tohoku.ac.jp; ytakahashi@tohoku.ac.jp

Supplementary Note 1: X-ray absorption spectroscopy

The macroscopic chemical properties of SPBMA were examined through X-ray absorption spectroscopy using the conversion electron yield (CEY) mode at the BL16 beamline of the SAGA Light Source (SAGA-LS, Saga, Japan). The X-ray absorption near-edge structure (XANES) at the sulfur *K*-edge was analyzed for the SPBMA powders using monochromatic X-rays scanned across the energy range 2455.1–2500.5 eV with 244 energy points, selected by a Si(111) double-crystal monochromator (*Δ*E/E ≈ 10⁻⁴). Figure S1 illustrates the X-ray absorption spectrum of SPBMA near the sulfur *K*-edge. The energy axis was calibrated so that the peak with the highest intensity appeared at 2471.1 eV, ensuring consistent alignment of spectra obtained at BL10U and BL16 for samples derived from the same batch. Two distinct peaks were identified near the white-line region around 2470 eV. Previous studies assigned these peaks through comparison with reference spectra^1^. The peak at higher energy with the maximum intensity was linked to the σ*(S–C) transition and is henceforth denoted as the S–C bonding peak. The peak at 2469.8 eV near the white line was associated with the σ*(S–S) transition and is labeled as the S–S bonding peak.

Figure S1. Sulfur K-edge X-ray absorption spectrum of SPBMA measured using the CEY mode. The left and right panels show the XANES region and the expanded view of the white-line region.

Supplementary Note 2: Scanning electron microscope with energy dispersive X-ray spectrometry

Scanning electron microscopy with energy-dispersive X-ray spectroscopy (SEM-EDX) was utilized to analyze the average composition of the SPBMA sample. The investigation was carried out using a focused ion beam scanning electron microscope (Scios, FEI Japan) equipped with an X-Max 150 detector (Oxford Instruments). Figure S2(a) displays an SEM image of SPBMA powders, with a field of view of approximately 650 µm (H) × 450 µm (V) containing numerous micrometer-sized particles. Subsequent to SEM observation, EDX measurements were conducted over the same field of view. Figure S2(b) illustrates the corresponding EDX spectrum. After excluding the low-energy peak attributed to electronic noise, the remaining peaks were identified as carbon, sulfur, or oxygen. The elemental mass fractions were determined from the spectrum. Table S1 summarizes the mass fractions of SPBMA obtained from the EDX analysis. Hydrogen was not considered in this analysis as it is not detectable by EDX. These mass fractions were calculated based on a significant number of particles within the measurement field, thus representing the average composition of the SPBMA sample.

Figure S2. (a) SEM image of SPBMA powders. and (b) EDX spectrum acquired from the same field of view, showing the full energy range (left) and an expanded view of the low-energy region (right).

Table S1. Average mass fraction of SPBMA obtained via EDX analysis.

| Carbon  (mass%) | Oxygen (mass%) | Sulfur  (mass%) |
| --- | --- | --- |
| 57.10 | 8.7 | 34.2 |

Supplementary Note 3: Ptychography measurement system

The ptychographic measurement system utilized in this study was slightly modified from previously reported designs^2^. In the adjusted setup, the mirror and sample chambers were directly connected and separated solely by a 37.5 µm-thick Kapton film to minimize attenuation of the incident X-ray flux at the sulfur *K*-edge. In the original configuration, attenuation by two 50 µm-thick Kapton films and a 10 mm air-filled ionization chamber restricted the X-ray intensity reaching the sample to approximately 3% of that reflected by the advanced Kirkpatrick–Baez (AKB) mirrors. In the modified arrangement, attenuation by a single 37.5 µm-thick Kapton film permits about 33% of the reflected X-ray intensity.

System performance was assessed using a 200 nm-thick Ta test chart, scanned in a raster pattern with a 500 nm step size across a 15 × 15 grid at 2500 eV. Each scan position had an exposure time of 1.0 s. Figure S3(a) illustrates a typical diffraction intensity pattern. Image reconstruction employed the extended ptychographic iterative engine (ePIE) algorithm with mixed-state reconstruction and position correction. The initial object had unit amplitude and zero phase, while the initial probe was a sinc function based on the beam size at the detector. A total of 500 iterations were conducted. The pixel size was 10.377 nm. Figure S3(b) displays the normalized intensity distribution of the reconstructed probe, with the first and second modes representing 94.2% and 5.8% of the total photon flux, respectively. Gaussian fitting of the intensity profile indicated a beam size of 1.48 µm (H) × 1.28 µm (V) using the full width at half maximum (FWHM). Figure S3(c) exhibits the reconstructed phase image of the test chart, revealing clear resolution of structures as small as 50 nm. Spatial resolution was further assessed by fitting edge profiles in the image. Figure S3(d) presents a line profile across an edge with the fitted error function. The average FWHM of the fitted profiles estimated the spatial resolution of the phase image to be approximately 20 nm.

Figure S3. (a) Diffraction pattern of the 200 nm-thick Ta test chart. (b) Intensity distributions of the reconstructed probe function for the first (left) and second (right) modes. (c) Reconstructed phase image of the Ta test chart. (d) Representative line profile extracted from an edge of the test chart, with the fitted full width at half maximum (FWHM).

Supplementary Note 4: SEM image

SEM images of the sample were obtained before the ptychographic measurements to analyze the particle morphology and sample positioning on the membrane. Figure S4 displays an SEM image of SPBMA particles situated on the flat area of the SiN membrane. The image represents a downstream perspective in the ptychographic measurement setup, with the SiN membrane window placed on the upstream side. This SEM image acts as a benchmark for assessing particle morphology pre-X-ray measurements against reconstructed ptychographic and PXCT images.

Figure S4. SEM image of SPBMA obtained prior to ptychographic measurements.

Supplementary Note 5: Fourier shell correlation and Fourier ring correlation

The spatial resolution was assessed using Fourier shell correlation (FSC) and Fourier ring correlation (FRC). FSC was computed from three-dimensional reconstructions at 2500 eV using two statistically independent projection datasets to evaluate isotropic spatial resolution. FRC was determined from two independently reconstructed projection images obtained at the same energy to assess in-plane spatial resolution. These metrics were employed to address the resolution resulting from the limited angular range of the PXCT measurements. The corresponding FSC and FRC curves are depicted in Figs. S5(a) and S5(b), respectively. The horizontal axis in these figures represents spatial frequency, which corresponds to the inverse of the real-space length scale. Higher frequencies probe finer structural details. The vertical axis indicates the correlation between two statistically independent reconstructions. Values approaching unity signify high structural similarity, while lower values suggest reduced structural similarity and increased noise.

Figure S5. Spatial resolution assessment using Fourier correlation analysis. (a) Fourier shell correlation (FSC) curves calculated from three-dimensional phase and absorption reconstructions at 2500 eV. The spatial resolution was determined using the 1/2-bit threshold criterion. (b) Fourier ring correlation (FRC) curves calculated from independently reconstructed phase and absorption projection images at 2500 eV. The in-plane spatial resolution was determined using the 1-bit threshold criterion.

Supplementary Note 6: Expected complex refractive index of SPBMA

The complex refractive index of the sample was determined based on the density of SPBMA (1.841 g cm⁻³) and the elemental mass fractions identified through EDX analysis (Table S1). The sample is heterogeneous, so the refractive index from this note should be treated as a rough estimate. In this analysis, only carbon, oxygen, and sulfur were the only elements considered in this evaluation. Hydrogen's contribution to the X-ray refractive index and absorption coefficient was disregarded due to its low atomic number and minimal impact near the sulfur *K*-edge. The mass of each element per unit volume was calculated by multiplying the sample density by the respective mass fraction. This resulting mass per unit volume was then divided by the atomic weight to derive the amount of substance, which was subsequently converted to atomic number density using Avogadro’s constant (6.022 × 10²³ mol⁻¹). The resulting atomic number densities are outlined in Table S2. By utilizing these densities and the atomic scattering factors at 2500 eV, the real and imaginary components of the refractive index of SPBMA were determined to be $\delta$=5.601 × 10⁻⁵ and $\beta$= 6.156 × 10⁻⁶, respectively. The atomic scattering factors were sourced from the Center for X-Ray Optics database^3^. The electron density of SPBMA was computed as 0.5078 electrons/Å³ by multiplying $\delta$ by the factor 2$\pi$/($r_{e}\lambda^{2}$), where $\lambda$ is the X-ray wavelength and *r*ₑ is the classical electron radius. The absorption coefficient at 2500 eV was calculated as $\mu$ = 4$\pi\beta$/$\lambda$ = 1.560 × 10⁵ m⁻¹. These values serve as the expected electron density and absorption of SPBMA, providing reference benchmarks for validating the quantitative PXCT results.

Table S2. Amounts of substance of each element per unit volume of SPBMA

|  | Carbon | Oxygen | Sulfur |
| --- | --- | --- | --- |
| Molar concentration (mol/cm^3^) | 0.08751 | 0.01003 | 0.01963 |
| Atomic number density (atoms/m^3^) | 5.270×10^28^ | 6.042×10^28^ | 1.182×10^28^ |

Supplementary Note 7: Practical signal to noise ratio

The signal-to-noise ratio (SNR) of $\Delta\mu t$ was evaluated to assess the reliability of $\Delta\mu t$ with respect to the noise sensitivity. In this study, noise was estimated based on the reproducibility of the reconstruction using a split-data approach, in which the measured data were divided into two subsets and reconstructed independently. The resulting $\Delta\mu t$ volumes from the two subsets, $\Delta{\mu t}_{half1}$ and $\Delta{\mu t}_{half2}$ were used to evaluate the noise component. The CT reconstruction procedure was identical to that described in the Methods section. Assuming that $\Delta\mu t$ consists of the true value $\Delta\mu t_{\text{truth}}$ with random noise terms $N_{1}$ and $N_{2}$, the two reconstructions can be expressed as:

$$\Delta{\mu t}_{half1}=\Delta\mu t_{\text{truth}}+N_{1},$$

$$\Delta{\mu t}_{half2}=\Delta\mu t_{\text{truth}}+N_{2}.$$

(S1)

Taking the difference between the two cancels the true value:

$$\Delta{\mu t}_{half1}-\Delta{\mu t}_{half2}=N_{1}-N_{2}.$$

(S2)

Let Var(·) and Cov(·,·) denote variance and covariance, respectively. Then,

$$\mathrm{Var}(N_{1}-N_{2})=\mathrm{Var}(N_{1})+\mathrm{Var}(N_{2})-2\text{ }\mathrm{Cov}(N_{1},N_{2}).$$

(S3)

Since both subsets were obtained from the same measurement system and have comparable statistical quality, the variances of $N_{1}$and $N_{2}$were assumed to be approximately equal. Furthermore, the noise terms were assumed to be independent, so the covariance term was neglected. Under these assumptions,

$$\mathrm{Var}(N_{1}-N_{2})\approx2\text{ }\mathrm{Var}(N_{1}).$$

(S4)

The standard deviation of the noise in the half datasets, $\sigma_{\mathrm{half}}$, is therefore given by

$$\sigma_{\mathrm{half}}\approx\sqrt{\mathrm{Var}(N_{1})}=\frac{\sqrt{\mathrm{Var}(\Delta\mu t_{\mathrm{half}1}-\Delta\mu t_{\mathrm{half}2})}}{\sqrt{2}}.$$

(S5)

This $\sigma_{\mathrm{half}}$ represents an effective noise metric reflecting random uncertainties arising from measurement and reconstruction in the half datasets. When averaging the two half datasets, the true value is preserved while random noise is partially canceled. Assuming that reconstructed$\Delta\mu$*t* from the full dataset corresponds to the average of two independent measurements,

$$\Delta\mu t_{\mathrm{full}}\approx\Delta\mu t_{\mathrm{truth}}+\frac{N_{1}+N_{2}}{2}.$$

(S6)

Using properties of variance,

$$\mathrm{Var}\left( \frac{N_{1}+N_{2}}{2} \right)=\frac{1}{4}\mathrm{Var}(N_{1}+N_{2}).$$

(S7)

Assuming independent noise terms and negligible covariance,

$$\frac{1}{4}\mathrm{Var}(N_{1}+N_{2})\approx\frac{1}{4}\mathrm{Var}(N_{1})+\frac{1}{4}\mathrm{Var}(N_{2})=\frac{\sigma_{\mathrm{half}}^{2}}{2}.$$

(S8)

Therefore, the standard deviation of the noise in the full dataset, $\sigma_{\mathrm{full}}$, is given by

$$\sigma_{\mathrm{full}}=\sqrt{\mathrm{Var}\left( \frac{N_{1}+N_{2}}{2} \right)}=\frac{\sigma_{\mathrm{half}}}{\sqrt{2}}.$$

(S9)

The noise standard deviation in the particle region $\sigma_{\mathrm{full}}$ was $2.73\times{10}^{-4}$, which was used as the noise term in the SNR. The signal component was defined using $\Delta\mu t_{\mathrm{full}}$. The SNR was defined as

$$\mathrm{SNR}=\frac{\Delta\mu t_{\mathrm{full}}}{\sigma_{\mathrm{full}}}.$$

(S10)

As a criterion for reliable signal discrimination from noise, a threshold of SNR $\geq$ 3 (corresponding to $\Delta\mu t_{\mathrm{full}}=8.19\times{10}^{-4}$) was adopted. This corresponds to the condition that the signal exceeds three times the standard deviation of the noise under a Gaussian assumption.

Supplementary Note 8: Particle identification and outlier detection of chemical states

To calculate the compositional parameters ($V_{\text{notS}}$, $V_{\text{SS}}$, and $V_{\text{SC}}$), sufficiently large $\Delta\mu t$ values were also identified using the Tukey fence. First, we define the domain for applying the Tukey fence to the volumetric data. Particle morphology and size were characterized using electron density. The particle and background regions were separated by applying Otsu’s method to the three-dimensional electron density dataset at 2500 eV, yielding a threshold of 0.220 electrons/Å³. Voxels identified as particles are denoted as the set ***E***. Otsu’s method was also applied to the absorption dataset at 2500 eV, resulting in a threshold of μt = 2.506 × 10⁻³. Voxels with μt values above this threshold are defined as the set ***M***.

The Tukey fence^4^ was applied to $\Delta\mu t$ values of voxels belonging to both ***E*** and ***M****.* Voxels with $\Delta\mu t$ ≤ 0 were excluded as noise. The analysis domain is defined as

$$\boldsymbol{\Omega}=\boldsymbol{E}\cap\boldsymbol{M}\cap\left\{ \boldsymbol{r} | \Delta\mu t\left( \boldsymbol{r} \right)>0 \right\},$$

(S11)

where $\boldsymbol{\Omega}$ represents the set of voxels subject to the Tukey-fence analysis. The lower fence $\Delta{\mu t}_{low}$ is given by

$$\Delta{\mu t}_{low}=Q_{1}-1.5\times IQR=2.103\times{10}^{-4},$$

(S12)

where $Q_{1}$ and *IQR* denote the first quartile and interquartile range of $\Delta\mu t$ in $\boldsymbol{\Omega}$, respectively.

Supplementary Note 9: Accessible information via PXCT

This Supplementary Note summarizes the physical quantities accessible by PXCT and presents the theoretical basis for the compositional parameters utilized in this study. In X-ray ptychography, the incident X-ray wavefield and the complex transmission function $T$ of the sample are reconstructed from measured diffraction patterns. The complex transmission function is given by

$$T\left( \boldsymbol{r}_{\boldsymbol{xz}}\boldsymbol{,}\lambda\right)=\exp\left( -\frac{2\pi}{\lambda}\int\beta\left( \boldsymbol{r}_{\boldsymbol{xz}}\boldsymbol{,}\lambda\right)dy \right)\exp\left( -i\frac{2\pi}{\lambda}\int\delta\left( \boldsymbol{r}_{\boldsymbol{xz}}\boldsymbol{,}\lambda\right)dy \right),$$

(S13)

where $\boldsymbol{r}_{\boldsymbol{xz}}$ denotes the position vector in the transverse plane, $x$ and$y$correspond to the horizontal and the vertical directions, respectively, and *y* denotes the optical axis. The parameters $\delta$ and $\beta$ are the real and imaginary parts of the complex refractive index, and $\lambda$ is the X-ray wavelength. The atomic scattering factor $f_{j}$and the complex refractive index $n$ are expressed as:

| $f_{j}=f_{j}^{0}+f_{j}^{'}+if_{j}^{''},$ | (S14) |  |
| --- | --- | --- |
| $n=1-\delta+i\beta,$  with | (S15) | |
| $\beta=\frac{r_{e}\lambda^{2}}{2\pi}\sum_{j} n_{j}f_{j}^{''},$ | (S16) | |
| $\delta=\frac{r_{e}\lambda^{2}}{2\pi}\sum_{j} n_{j}\left( f_{j}^{0}+f_{j}^{'} \right)\cong\frac{r_{e}\lambda^{2}}{2\pi}\sum_{j} n_{j}\left( Z_{j}+f_{j}^{'} \right),$ | (S17) | |

where $r_{e}$ is the classical electron radius, $n_{j}$ is the number density of species $j$, and $Z_{j}$ is the atomic number. In this study, the scattering angle satisfies $\theta\leq0.024$ rad, which is sufficiently small to justify the forward-scattering approximation. Accordingly, the scattering vector $\boldsymbol{k}(=\frac{4\pi}{\lambda}\sin\theta)$ can be approximated as $\boldsymbol{k}\cong0$, and the atomic scattering factor as $f_{j}^{0}\left( \boldsymbol{k} \right)\cong Z_{j}$. Under these approximations, the phase image $\Phi\left( \boldsymbol{r,}\lambda\right)$ and the absorption image $\mu t\left( \boldsymbol{r,}\lambda\right)$ are expressed as

$$\Phi\left( \boldsymbol{r}_{\boldsymbol{xz}},\lambda\right)=\arg\left\{ T\left( \boldsymbol{r}_{\boldsymbol{xz}}\boldsymbol{,}\lambda\right) \right\}$$

$$=-\frac{2\pi}{\lambda}\int\delta\left( \boldsymbol{r}_{\boldsymbol{xz}}\boldsymbol{,}\lambda\right)dy$$

$$=\frac{4\pi}{\lambda}\cdot\frac{r_{e}\lambda^{2}}{2\pi}\int\sum_{j} n_{j}\left( \boldsymbol{r}_{\boldsymbol{xz}} \right)\cdot\left\{ Z_{j}+f_{j}^{'}\left( \lambda\right) \right\}dy\text{,}$$

(S18)

$$\mu t\left( \boldsymbol{r}_{\boldsymbol{xz}}\boldsymbol{,}\lambda\right)=-2\cdot log \left| T\left( \boldsymbol{r}_{\boldsymbol{xz}}\boldsymbol{,}\lambda\right) \right|$$

$$=\frac{4\pi}{\lambda}\int\beta\left( \boldsymbol{r}_{\boldsymbol{xz}}\boldsymbol{,}\lambda\right)dy$$

$$=\frac{4\pi}{\lambda}\cdot\frac{r_{e}\lambda^{2}}{2\pi}\int\sum_{j} n_{j}\left( \boldsymbol{r}_{\boldsymbol{xz}} \right)\cdot f_{j}^{''}(\lambda)dy.$$

(S19)

CT reconstruction of the phase and absorption projections yields three-dimensional distributions as follows:

$$\Phi\left( \boldsymbol{r},\lambda\right)=-\frac{2\pi}{\lambda}\delta\left( \boldsymbol{r,}\lambda\right)t,$$

(S20)

$$\mu t\left( \boldsymbol{r}, \lambda\right)=\frac{4\pi}{\lambda}\beta(\boldsymbol{r},\lambda)t,$$

(S21)

where *t* denotes the voxel edge length. At X-ray energies far from elemental absorption edges, the dispersion correction term $f'$ is negligible, and the electron density distribution $\rho_{ED}$ can be obtained as:

$$\rho_{\mathrm{ED}}=\frac{2\pi}{r_{e}\lambda^{2}}\delta\left( \boldsymbol{r},\lambda\right)=-\frac{2\pi}{r_{e}\lambda^{2}}\cdot\frac{\lambda}{2\pi t}\Phi\left( \boldsymbol{r},\lambda\right).$$

(S22)

PXCT performed near an elemental absorption edge enables the spatial mapping of the corresponding element. When the absorption $\mu t\left( \boldsymbol{r}, \lambda_{\text{post}} \right)$and $\mu t\left( \boldsymbol{r}, \lambda_{\text{pre}} \right)$ are acquired at wavelengths above and below the sulfur *K*-edge, respectively, their difference is approximately proportional to the sulfur atomic number density $n_{\text{S}}(\boldsymbol{r})$.

$$\Delta\mu t= \mu t\left( \lambda_{\text{post}} \right)-\mu\left( \lambda_{\text{pre}} \right)$$

$$=4\pi t\left\{ \frac{\beta\left( \lambda_{\text{post}} \right)}{\lambda_{\text{post}}}-\frac{\beta\left( \lambda_{\text{pre}} \right)}{\lambda_{\text{pre}}} \right\}$$

$$=4\pi t\left\{ \frac{r_{e}\lambda_{\text{post}}}{2\pi}\sum_{j} n_{j}\cdot f_{j}^{''}\left( \lambda_{\text{post}} \right)-\frac{r_{e}\lambda_{\text{pre}}}{2\pi}\sum_{j} n_{j}\cdot f_{j}^{''}\left( \lambda_{\text{pre}} \right) \right\}$$

$$\approx2r_{e}t\lambda_{\text{post}}\cdot n_{\text{S}}\cdot\{f_{\text{S}}^{''}\left( \lambda_{\text{post}} \right)-f_{\text{S}}^{''}\left( \lambda_{\text{pre}} \right)\}$$

(S23)

This approximation holds true due to the minimal variation in atomic scattering factors of elements besides sulfur around the sulfur *K*-edge, and $\lambda_{\text{post}}\cong\lambda_{\text{pre}}$ in the current experiment. Thus, we define $V_{\text{notS}}$as the electron density normalized by $\Delta\mu$.

$$V_{\text{notS}}= \frac{\rho_{ED}}{\Delta\mu}$$

$$\approx\frac{\sum_{j} n_{j}\left\{ Z_{j}+f_{j}^{'}\left( \lambda_{\text{post}} \right) \right\}}{2r_{e}\lambda_{\text{post}}t\cdot n_{\text{S}}\cdot\{f_{\text{S}}^{''}\left( \lambda_{\text{post}} \right)-f_{\text{S}}^{''}\left( \lambda_{\text{pre}} \right)\}}$$

$$\propto\frac{n_{\text{S}}}{n_{\text{S}}}Z_{S}+\frac{n_{\text{C}}}{n_{\text{S}}}Z_{\text{C}}+\frac{n_{\text{O}}}{n_{\text{S}}}Z_{\text{O}}$$

(S24)

The relative contribution of non-sulfur elements is reflected in SPBMA. Since SPBMA is primarily consists of sulfur, carbon, and oxygen, higher values of $V_{\text{notS}}$ indicate areas with higher carbon or oxygen content compared to sulfur. Additionally, the absorption images measured near the S–C and S–S bonding peaks, ${\mu t}_{\text{SC}}$ and ${\mu t}_{\text{SS}}$, respectively, are directly related to the quantities of sulfur present in S–C and S–S bonds. This relationship defines the following:

$$V_{\mathrm{SC}}=\frac{{\mu t}_{\mathrm{SC}}-{\mu t}_{\mathrm{post}}}{\Delta\mu t}$$

(S25)

$$V_{\mathrm{SS}}= \frac{{\mu t}_{\mathrm{SS}}-{\mu t}_{\mathrm{post}}}{\Delta\mu t}$$

(S26)

The subtraction of ${\mu t}_{\mathrm{post}}$ defines the peak intensities, division by $\Delta\mu t$ normalizes the sulfur concentration, enabling the comparison of sulfur-bonding states independent of total sulfur concentration.

Supplementary Note 10 : Shape-based particle segmentation

Particles were categorized into spherical and non-spherical regions based on their morphology. Initially, an Euclidean distance transform was utilized on the binarized particle mask to determine the distance of each voxel from the nearest background voxel^5^. Subsequently, local maxima in the distance map were identified as potential particle centers. These local maxima were detected using a threshold set at 0.5% of the maximum distance from the particle surface. Watershed segmentation was then executed by adjusting the distance values at the marker positions to local minima^6^. This method facilitated the separation of particles by creating dividing lines at their contact regions, thereby resolving touching particles.

After watershed segmentation, connected-component analysis with 26-neighbor connectivity was utilized on the volumetric data to assign a unique integer label to each particle. Geometric characteristics of each labeled region, such as particle volume $V$, surface area $A$, and principal axis lengths, were then extracted. The sphericity $S_{p}$ and the principal-axis ratio $l_{\mathrm{ratio}}$ were defined based on these parameters as follows:

$$S_{p}=\pi^{1/3}\frac{\left( 6V)^{2/3} \right.}{A}$$

(S27)

$$l_{ratio}=\frac{L_{\text{max}}}{L_{\text{min}}}$$

(S28)

Here, $L_{\text{max}}$ and $L_{\text{min}}$ denote the maximum and minimum principal axis lengths, respectively. The sphericity $S_{p}$ equals 1 for a perfect sphere and decreases as the particle shape deviates from sphericity. The principal-axis ratio $l_{\mathrm{ratio}}$ serves as a measure of shape anisotropy, with values closer to 1 indicating more isotropic shapes. In this study, regions within the particle satisfying $S_{p}\geq0.8$ and $l_{\mathrm{ratio}}\leq1.1$ were defined as spherical regions. These thresholds were selected to match visual classification, and voxels outside these criteria were classified as non-spherical regions.

Supplementary Note 11: X-ray spectroscopic ptychography of several particles

X-ray spectroscopic ptychography measurements were conducted on multiple particles prepared similarly to those utilized in the PXCT experiments. During the allocated beamtime, the measurements were replicated, and four fields of view were captured in this investigation. The virtual source size was adjusted to 10 µm (H) × 100 µm (V), and diffraction patterns were gathered at 30 energy points spanning 2455.0–2500.0 eV near the sulfur *K*-edge. The focal spot size was 1.48 µm (H) × 1.28 µm (V). For each energy point, the sample underwent raster scanning with a 500 nm step size, and the exposure time was 0.5 s per scan position. Subsequently, sample images were reconstructed from the diffraction patterns using mixed-state ePIE with 800 iterations. The probe wavefield derived from the test-chart measurement served as the initial probe function, while a uniform object with unit amplitude and zero phase was utilized as the initial object function. Initially, the absorption image was guided by the phase image to enhance convergence during the first 300 iterations. The pixel size for all reconstructed images was standardized to 25 nm. Figure S6(a) illustrates the phase images of the four fields of view, with the particles in each field labeled as P1, P2, P3, and P4. Notably, particle P2 appears predominantly spherical, whereas P1, P3, and P4 exhibit largely non-spherical morphologies.

Chemical state distributions were analyzed by calculating the edge jump (EJ), the EJ-normalized phase shift, and the EJ-normalized S–S and S–C peak intensities. Due to differences in measurement energies compared to PXCT particles, the energy ranges and sampling points for absorption averaging were redefined (Table S3). Figures S6(b)–S6(e) display the EJ, the EJ-normalized phase shift, and the EJ-normalized S–S and S–C peak intensities for the SPBMA particles. Spatial variations were observed in all parameters among and within individual particles. To quantify these variations, the median and quartiles of each parameter were calculated and summarized using box-and-whisker plots. Figure S6(f) illustrates the box plots and interquartile ranges of the four parameters for the four fields of view. The EJ-normalized S–C peak intensity exhibited relatively uniform values within each particle. Comparing parameters based on particle morphology, the spherical particle P2 showed smaller interquartile ranges for all parameters than the non-spherical particles. Additionally, P2 displayed a notably higher EJ-normalized S–S peak intensity, while non-spherical particles P1, P3, and P4 had lower S–S peak intensities. These findings suggest that spherical particles contain a relatively higher amount of sulfur involved in S–S bonding.

Table S3. Energy ranges and sampling points used for absorption averaging in chemical-state mapping of multiple particles

| Averaged absorption | Energy range (eV) | Energy points |
| --- | --- | --- |
| $\left\langle{\mu t}_{\text{post}} \right\rangle_{E}$ | 2492.0－2500.0 | 3 |
| $\left\langle{\mu t}_{\text{pre}} \right\rangle_{E}$ | 2455.0－2464.5 | 4 |
| $\left\langle{\mu t}_{\text{SS}} \right\rangle_{E}$ | 2469.3－2470.3 | 4 |
| $\left\langle{\mu t}_{\text{SC}} \right\rangle_{E}$ | 2407.0－2471.0 | 2 |

Figure S6. Chemical-state maps of SPBMA particles P1–P4. (a) Phase images, (b) edge jump (EJ), (c) EJ-normalized phase shift, (d) EJ-normalized S–S peak intensity, and (e) EJ-normalized S–C peak intensity, shown from left to right for particles P1–P4. (f) Box-and-whisker plots of the four parameters for each particle. In panels (b–e), pixels with EJ < 0.10 are shown in white, with particle boundaries shown in black. Particle regions were extracted by k-means clustering of the phase images.

Supplementary Note 12: Radiation dose

The radiation dose $D$delivered to the sample was calculated as follows^7^:

$$D=\mu_{sample}\frac{F_{0}E}{\rho},$$

(S29)

where $\mu_{sample}$is the absorption coefficient of the sample (m⁻¹), $F_{0}$ is the photon fluence (photons m⁻²), $E$ is the X-ray energy (J), and $\rho$ is the sample density (kg m⁻³). The radiation dose is expressed in gray (Gy = J kg⁻¹).

First, the photon fluence was calculated in the experiment. The sample was positioned downstream of a SiN membrane. Let $I_{0}$ be the incident photon flux on the 200 nm-thick SiN membrane, and $I_{0}^{\mathrm{SiN}}$be the transmitted flux after attenuation by the membrane. Assuming normal incidence (0°) of the X-ray beam on the membrane and an absorption coefficient of the SiN membrane $\mu_{\mathrm{SiN}}$ (m⁻¹), the following relation holds:

$$I_{0}^{\mathrm{SiN}}=I_{0}\cdot\exp\left\{ -\mu_{SiN}t_{0} \right\},$$

(S30)

where $t_{0} (=200 \text{nm})$ is the membrane thickness. Owing to the increase in effective thickness of the SiN membrane along the X-ray path with the incident angle $\theta$, the incident flux is attenuated correspondingly. At an incident angle $\theta$, the effective membrane thickness becomes $t_{0}$/$\cos\theta$. The photon flux incident on the sample after transmission through the SiN membrane at angle $\theta$, denoted as $I_{0}^{\text{sample}}$, is thus given by

$I_{0}^{sample}\left( \theta,E \right)= I_{0}\cdot\exp\left\{ -\mu_{SiN}\left( E \right)\frac{t_{0}}{\cos\theta} \right\}$.

(S31)

In the experiment, the photon flux $I_{0}^{\mathrm{SiN}}$ at each energy was measured using the CITIUS detector, which accounted for the attenuation by the 5 μm-thick Kapton window^2^. Rearranging the above expressions yields.

$$I_{0}^{sample}\left( \theta,E \right)=\frac{I_{0}^{SiN}}{\alpha\cdot\exp\left\{ {-\mu}_{SiN}\left( E \right)\cdot t_{0} \right\}}\exp\left\{ -\mu_{SiN}\left( E \right)\frac{t_{0}}{\cos\theta} \right\}$$

$$=\frac{I_{0}^{SiN}\left( E \right)}{\alpha}\cdot\exp\left\{ -\mu_{SiN}(E)\cdot t_{0}\cdot\left( \frac{1}{\cos\theta}-1 \right) \right\}$$

(S32)

where $E$ denotes the photon energy, and $\alpha$denotes the transmission of the 5 μm-thick Kapton film. In this study, projection images were acquired at 113 equally spaced incidence angles over a range of ±84°. At 2500 eV, the beam size was 1.48 µm (H) × 1.28 µm (V). In ptychographic measurements, the sample is scanned with a probe having an incident intensity $I_{0}^{sample}$. The region of interest for photon fluence was defined as the area irradiated by the main probe beam at least four times. Under this definition, the photon fluence per ptychographic scan is given by

$$F_{0}\left( \theta,E \right)=\frac{P_{\mathrm{total}} \left( E, \theta\right)}{A^{\mathrm{Pty}}\left( E \right)},$$

(S33)

where $P_{\text{total}}$ denotes the total number of photons within the region of interest, and $A^{\text{Pty}}$ denotes the area irradiated by the main probe beam at least four times. For CT measurements, this contribution is summed over all projection angles and energies. Accordingly, the radiation dose for CT, $D_{\text{CT}}$, is calculated as

$$D_{CT}=\sum_{E} \sum_{\theta} \left\{ \mu_{sample}\left( E \right)\cdot\frac{E}{\rho}\cdot F_{0}(\theta,E) \right\}.$$

(S34)

The radiation dose delivered to the sample was calculated. Table S4 summarizes the dose applied during the CT measurements, along with the X-ray flux, photon fluence, absorption coefficient, and density used in the calculations. The exposure time per ptychographic scan position was set to 0.5 s, and the photon fluence was calculated considering beam overlap. The absorption coefficient of the sample was determined as the median value of the reconstructed absorption coefficient distribution. For the complete CT dataset consisting of 113 projection angles and four energy points, the total radiation dose delivered to the sample surface was estimated to be 5.18  ×  10⁹ Gy.

Table S4. Radiation dose in CT measurements and the parameters used for its calculation.

| Photon energy (eV) | 2463.0 | 2469.6 | 2470.7 | 2500 |
| --- | --- | --- | --- | --- |
| Photon energy (J) | 3.946×10^-16^ | 3.957×10^-16^ | 3.958×10^-16^ | 4.005×10^-16^ |
| Incident flux $I_{0}$(photons/s) | 9.6×10^8^ | 9.57×10^8^ | 1.0×10^9^ | 9.7×10^8^ |
| Fluence at normal incidence $0^{\circ}$  $F_{0}$(photons/m^2^) | 2.0×10^20^ | 2.0×10^20^ | 2.1×10^20^ | 2.3×10^20^ |
| Absorption coefficient  $\mu$(m^-1^) | 0.287×10^5^ | 3.87×10^5^ | 4.62×10^5^ | 2.06×10^5^ |
| Density  $\rho$(kg/m^3^) | 1841 | 1841 | 1841 | 1841 |
| Dose of one  PXCT scan (Gy) | 1.31×10^8^ | 1.77×10^9^ | 2.21×10^9^ | 1.07×10^9^ |

**References**

1.	Abe, M. *et al.* Visualization of Sulfur Chemical State of Cathode Active Materials for Lithium–Sulfur Batteries by Tender X-ray Spectroscopic Ptychography. *J. Phys. Chem. C* **126**, 14047–14057 (2022).

2.	Sasaki, Y. *et al.* Broadband high-resolution X-ray ptychography system spanning tender to hard X-ray regimes. *IUCrJ* **13**, (2026).

3.	Henke, B. L., Gullikson, E. M. & Davis, J. C. X-Ray Interactions: Photoabsorption, scattering, transmission, and reflection at E = 50-30,000 eV, Z = 1-92. *Atomic Data and Nuclear Data Tables* **54**, 181–342 (1993).

4.	Tukey, J. W. *Exploratory Data Analysis*. (Addison-Wesley publ, Reading (Mass.) Menlo Park (Calif.) London [etc.], 1977).

5.	Maurer, C. R., Rensheng Qi & Raghavan, V. A linear time algorithm for computing exact Euclidean distance transforms of binary images in arbitrary dimensions. *IEEE Trans. Pattern Anal. Machine Intell.* **25**, 265–270 (2003).

6.	Meyer, F. Topographic distance and watershed lines. *Signal Processing* **38**, 113–125 (1994).

7.	Howells, M. R. *et al.* An assessment of the resolution limitation due to radiation-damage in X-ray diffraction microscopy. *J. Electron Spectrosc. Relat. Phenom.* **170**, 4–12 (2009).
